# Molecular chirality mediated amyloid formation on phospholipid surfaces†

**DOI:** 10.1039/d0sc02212h

**Published:** 2020-06-25

**Authors:** Xue Wang, Cunli Wang, Huiying Chu, Haijuan Qin, Dongdong Wang, Feifei Xu, Xuanjun Ai, Chunshan Quan, Guohui Li, Guangyan Qing

**Affiliations:** State Key Laboratory of Advanced Technology for Materials Synthesis and Processing, Wuhan University of Technology 122 Luoshi Road Wuhan 430070 P. R. China; Key Laboratory of Separation Science for Analytical Chemistry, Dalian Institute of Chemical Physics, Chinese Academy of Sciences 457 Zhongshan Road Dalian 116023 P. R. China qinggy@dicp.ac.cn; Laboratory of Molecular Modeling and Design, State Key Laboratory of Molecular Reaction Dynamics, Dalian Institute of Chemical Physics, Chinese Academy of Sciences 457 Zhongshan Road Dalian 116023 P. R. China ghli@dicp.ac.cn; Research Centre of Modern Analytical Technology, Tianjin University of Science and Technology Tianjin 300457 P. R. China; College of Life Science, Dalian Minzu University Dalian 116600 P. R. China

## Abstract

One of the neuropathological features of Alzheimer's disease (AD) is the misfolding of amyloid-β to form amyloid aggregates, a process highly associated with biological membranes. However, how molecular chirality affects the amyloid formation on phospholipid surfaces has seldom been reported. Here, l- and d-aspartic acid-modified 1,2-dipalmitoyl-*sn*-glycero-3-phosphoethanolamine (l-/d-Asp–DPPE) is synthesized to construct chiral phospholipid bilayers. We discover that the l-Asp–DPPE liposomes slightly inhibit the Aβ(1–40) nucleation process but cannot affect the oligomer elongation process. By contrast, the d-Asp–DPPE liposomes strongly inhibit both nucleation and elongation of the peptide. Notably, l- and d-Asp–DPPE liposomes not only have good biocompatibility but can also rescue Aβ(1–40)-aggregation induced cytotoxicity with significant chiral discrimination, in which the cell viability is higher in the presence of d-Asp–DPPE liposomes. Mechanism analysis and molecular dynamics simulation clearly demonstrate that differential electrostatic interactions of Lys16 in Aβ(1–40) with l- or d-Asp on the phospholipid contribute to the remarkable chiral discrimination. This study provides a deeper understanding of the crucial amyloidosis process from the perspective of the chiral interface and reveals that the convergence of d-amino acids with the liposomes might be a feasible route for AD prevention.

## Abstract 2

A remarkable inhibition effect and chiral discrimination are observed when the amyloid peptide aggregates on chiral phospholipid surfaces.

## Graphical abstract

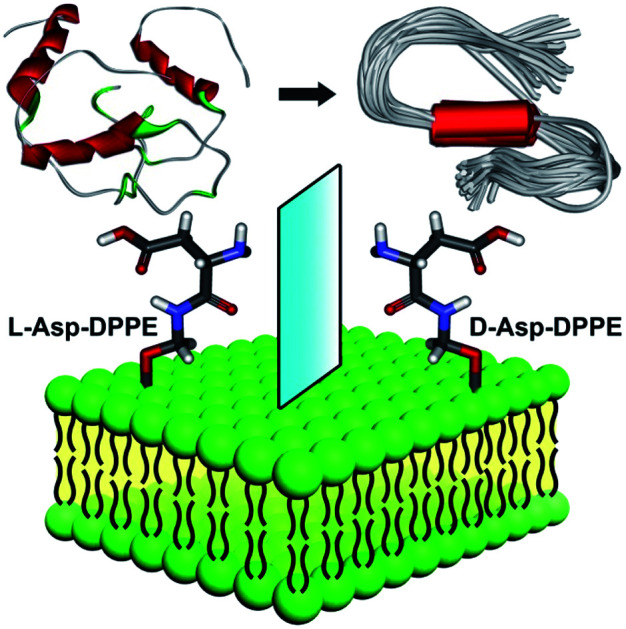

## Introduction

Alzheimer's disease (AD), the most common form of dementia, is a typically progressive neurodegenerative pathology of the central nervous system^1^ and has become one of the biggest global public health challenges facing our generation.^2^ As a protein-misfolding disease, the main neuropathological characteristic of AD is the accumulation of amyloid-β (Aβ) in plaques within extracellular spaces and the walls of blood vessels.^3^ Further insight into the pathophysiology of AD revealed that the plasma membrane of nerve cells plays a crucial role in the progress of AD.^4^ Thus, investigating the effect of molecular interfaces, particularly biological membranes, on amyloid formation is essential.

Most previous studies have reported the effect on amyloid formation of the properties of phospholipid membranes, such as their composition,^5^ hydrophobicity–hydrophilicity,^6^ charge,^7^ curvature,^8^ rafts,^9^ and GM1,^10^ and have also demonstrated the mechanism of membrane disruption by Aβ.^11^ However, researchers ignored an important issue, that is, the natural phospholipid is a chiral molecule showing very high preference to the l-enantiomer.^12^ More importantly, life is a typical chiral system and chiral phenomena are ubiquitous in nature from molecular, macromolecular, and supramolecular to macroscopic levels. Countless biological and physiological processes are closely related to molecular chirality,^13^ while the chiral preference is one important parameter for the study of the origin of life. This strongly inspired researchers to investigate how the molecular chirality of the phospholipid membrane impacts the Aβ aggregation process.

**Scheme 1:**
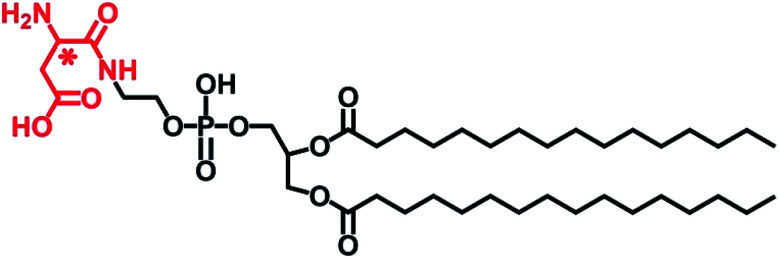
Chemical structure of l- or d-Asp-modified DPPE studied in this work, abbreviated to l- or d-Asp–DPPE.

In this respect, our group, Qu and others used a series of artificial materials, such as graphene,^14^ gold,^15^ polyoxometalate,^16^ mica wafers,^17^ carbon dots,^18^ and SiO_2_ (ref. 19), to construct chiral interfaces and preliminarily demonstrated that amino acid chirality could influence the fibrillation process of amyloid, which might offer new strategies for possible therapeutic avenues addressing protein-misfolding diseases. However, it is well known that the cell membrane is composed of a phospholipid bilayer. When Aβ comes into contact with the cell membrane, the phospholipid bilayers not only provide a supporting surface for Aβ to promote its adsorption, but might also influence the stereo-configuration of the amino acids and even the conformational transition of Aβ peptide through the strong chiral environment originating from the highly ordered assembly of the l-phospholipid. Further observation reveals that the cell membrane is covered with abundant chiral biomolecules, such as amino acids, peptides, proteins, and glycans, which are either inserted into the phospholipid bilayers or modified on the head end of the phospholipid molecules. This constructs an ideal chiral biointerface for the interaction with Aβ peptide. To mimic this feature, for the first time, we design a pair of chiral amino acid-modified phospholipid molecules and demonstrate the remarkable influence of the molecular chirality of chiral liposomes on amyloid formation (Scheme 1 and S1 in the ESI†).

In this design, amino acids were chosen as chiral moieties to modify the phospholipid, because the amino acids that constitute living organisms are mostly levorotatory. However, d-amino acids have been detected in various higher-level organisms in the form of free amino acids, peptides, and proteins, whereas l-amino acids can be converted into their d-enantiomers in the presence of various isomerases.^20^ Notably, the racemization of the aspartic acid (Asp) residues of Aβ peptide affects fibril formation,^21^ while d-Asp plays crucial roles in the nervous and neuroendocrine systems and is closely related to the development of AD.^22^ On the other hand, phosphoethanolamines (PEs) are one of the components of the neuronal membrane.^23^ Therefore, l- or d-Asp was reacted with the terminal amine of 1,2-dipalmitoyl-*sn*-glycero-3-phosphoethanolamine (DPPE) through a condensation reaction (Scheme S2 in the ESI†), preparing a pair of l- or d-Asp-modified phospholipids (abbreviated to l- or d-Asp–DPPE). When the self-assembled l- or d-Asp–DPPE liposomes were incubated with Aβ(1–40) monomers or oligomers, respectively, significant chiral discrimination in amyloid formation was observed on the phospholipid membranes. Meanwhile, cytotoxicity study demonstrated well the biocompatibility of the prepared chiral liposomes and their remarkable chiral discrimination in the ability to rescue Aβ(1–40)-aggregation induced cytotoxicity. Furthermore, adsorption dynamics and affinity tests showed that d-Asp–DPPE liposomes had a stronger adsorption and binding capacity for Aβ(1–40) compared with l-Asp–DPPE liposomes. In view of this, ^1^H–^15^N heteronuclear single quantum coherence (HSQC) nuclear magnetic resonance (NMR) spectra were collected to reveal the possible binding sites of Aβ(1–40) on the chiral phospholipid surface. Finally, their binding details were clearly displayed by molecular dynamics simulation. The novelty of this paper consists in providing a new perspective for investigating the relationship between the molecular chirality of biological membranes and amyloid formation and revealing that non-natural amino acid-modified phospholipids may be employed in the prevention of AD.

## Results and discussion

### Characterization of chiral phospholipid molecules and chiral liposomes

**Fig. 1:**
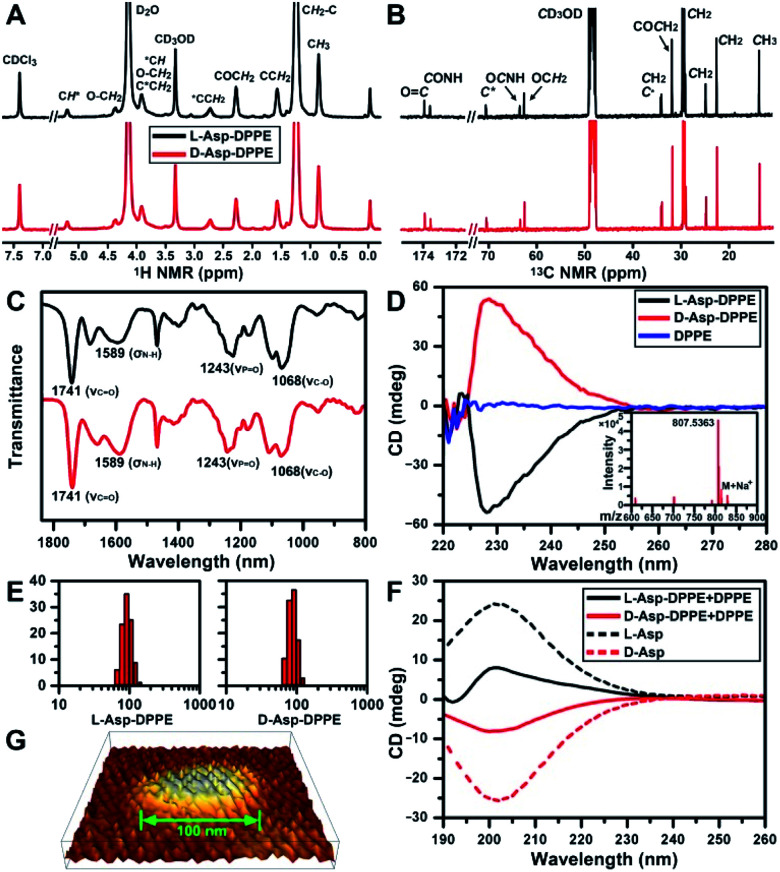
Characterization of chiral phospholipid molecules and chiral liposomes. (A and B) ^1^H (A) and ^13^C NMR (B) spectra of l- and d-Asp–DPPE in a CDCl_3_/CD_3_OD/D_2_O (v/v/v: 90 : 5 : 5) mixture. (C) FT-IR spectra of l- and d-Asp–DPPE. (D) CD spectra of l- and d-Asp–DPPE and DPPE in a CHCl_3_/CH_3_OH mixture (v/v: 3 : 1, 2 mg mL^−1^). The inset of (D) shows the MS of l-Asp–DPPE. (E and F) Hydrodynamic radius distribution (E) and CD spectra (F) of l- and d-Asp–DPPE liposomes mixed with an equal mass of DPPE in phosphate buffer solution (1 mg mL^−1^) at 25 °C; (F) CD spectra of l- and d-Asp in H_2_O (1 mg mL^−1^) at 25 °C, indicated by short dashed lines. (G) Three-dimensional image of a d-Asp–DPPE liposome obtained using AFM.

The structure of l- or d-Asp–DPPE was confirmed using ^1^H and ^13^C NMR spectra, Fourier transform infrared (FT-IR) spectra, and mass spectra (MS) (Fig. 1A–C and inset in D). The characteristic peaks and their chemical attributes are indicated in the figures. First, the synthesized chiral phospholipid molecules were detected in the circular dichroism (CD) spectra. The CD spectra of l- and d-Asp–DPPE were mirror-symmetry centered at 228 nm, and the chiral signal of the individual DPPE was negligible (Fig. 1D). Subsequently, l- or d-Asp–DPPE was mixed with DPPE in a mass ratio of 1 : 1, and stable liposomes with a uniform particle size of 100 nm were obtained through a classical extrusion method,^24^ the apparatus of which is shown in Fig. S1 in the ESI.† An equal mass of DPPE was added in order to improve the stability of the liposomes in the subsequent tests. The results of dynamic light scattering (DLS) and atomic force microscopy (AFM) demonstrated that uniform liposomes with an average diameter of 100 nm were formed (Fig. 1E and G). Furthermore, the CD spectra revealed that the prepared chiral liposomes had favorable mirror symmetry and the chirality was inherited from l- or d-Asp (Fig. 1F).

### Influence of chiral Asp-modified liposomes on the fibrillation process of Aβ(1–40)

**Fig. 2:**
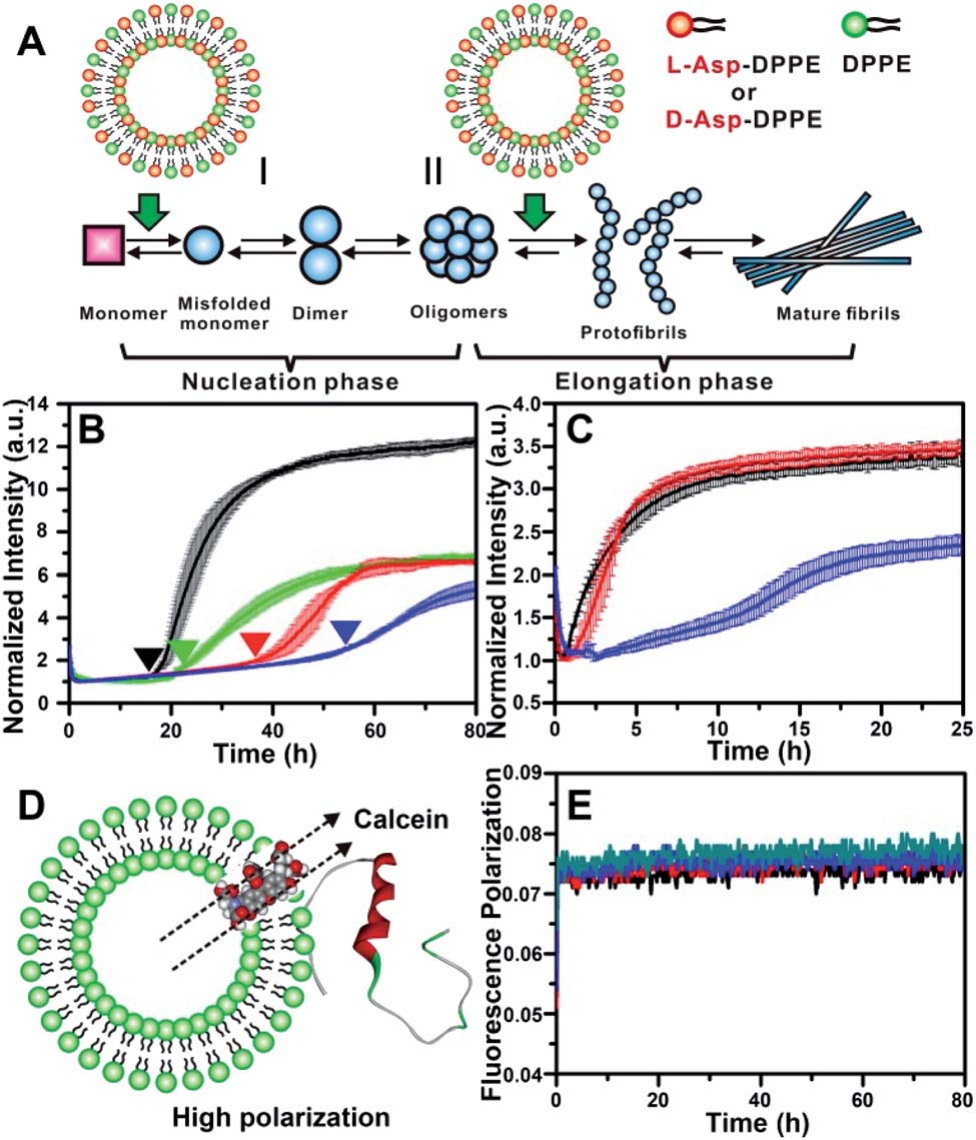
Influence of l-/d-Asp–DPPE liposomes on the fibrillation process of Aβ(1–40). (A) Two stages of fibrillation of Aβ(1–40) peptide, the nucleation and elongation phases.^26^ (B) ThT monitored kinetic curves for fiber formation of Aβ(1–40) monomers at 37 °C: Aβ(1–40) alone (black); Aβ(1–40) with l- (red), d-Asp–DPPE (blue), or DPPE (green) liposomes added. (C) ThT monitored kinetic curves for fiber formation of Aβ(1–40) oligomers at 37 °C: Aβ(1–40) alone (black); Aβ(1–40) with l- (red) or d-Asp–DPPE (blue) liposomes added. In all panels of (B) and (C), error bars show the standard deviations of the averaged data sets. Experiments were performed three times. (D) Schematic diagram of Aβ(1–40) interacting with calcein-loaded liposomes, corresponding to a strong fluorescence polarization due to the restriction of free rotation of calcein. (E) Calcein monitored fluorescence polarization kinetic curves: l- (black) or d-Asp–DPPE liposomes alone (blue); l- (red) or d-Asp–DPPE liposomes (green) incubated with Aβ(1–40) monomers at 37 °C. In these experiments, the final concentrations of Aβ(1–40) and chiral liposomes are 25 μM and 0.5 mg mL^−1^, respectively. 1% DMSO was added to the solution in order to improve the solubility of the peptide.

The most common isoforms of Aβ are Aβ(1–40) and Aβ(1–42), of which Aβ(1–40) is the predominant species and regarded as the classic pathological model.^25^ Thus, Aβ(1–40) was selected as the research object here. Considering the neurotoxicity of Aβ(1–42), we will discuss it in other work. The fibrillation process of the Aβ(1–40) peptide can be mainly divided into nucleation and elongation phases,^26^ as illustrated in Fig. 2A. We first investigated the influence of l- or d-Asp–DPPE liposomes on the nucleation phase of Aβ(1–40). In this experiment, one of various liposomes (1 mg mL^−1^) was added to an equal volume of Aβ(1–40) solution (50 μM in phosphate buffer solution) at the beginning of incubation at 37 °C; the rate of amyloid formation was then monitored using the standard thioflavin-T (ThT) binding assay.^27^ According to the growth curve of Aβ(1–40), as shown by the black line in Fig. 2B, Aβ(1–40) began to aggregate at 18 h and was completely fibrillated after 40 h, which was consistent with previous studies.^14,28^ The fibrillation of Aβ(1–40) was not affected when l- or d-Asp small molecules were added (Fig. S2 in the ESI†). As shown in Fig. 2B, the addition of DPPE liposomes (green curve) had a moderate inhibition effect on Aβ(1–40) aggregation, reflected in a clear decrease of the ThT fluorescence signal, and the aggregation time of Aβ(1–40) was delayed from 18 h to 22 h, which was confirmed by AFM observation (Fig. S6B in the ESI†). Interestingly, after adding l-Asp–DPPE liposomes (red curve), Aβ(1–40) began to aggregate at 36 h, which was 18 h later than Aβ(1–40) alone. By comparison, adding d-Asp–DPPE liposomes (blue curve) resulted in postponement of Aβ(1–40) aggregation to 54 h, at which time fibrosis of Aβ(1–40) was complete for the addition of l-Asp–DPPE liposomes. This result indicated that the addition of l-Asp–DPPE liposomes slightly inhibited the fibrillation of Aβ(1–40), whereas the addition of d-Asp–DPPE liposomes strongly inhibited this process. Moreover, no evident change was observed in the size of the liposomes during the peptide incubation process detected by a DLS test (Fig. S3 in the ESI†), revealing that liposomes infrequently aggregated or collapsed when they interacted with the peptides.

To study the influence of chiral liposomes on the fiber elongation phase, l- or d-Asp–DPPE liposomes were added to the Aβ(1–40) peptide solution after 18 h of incubation at 37 °C, at which time peptide oligomers had formed. As shown in Fig. 2C, the addition of l-Asp–DPPE liposomes (red curve) had little effect on the fiber elongation process. However, when d-Asp–DPPE liposomes were added (blue curve), the growth of Aβ(1–40) oligomers was inhibited, and elongation began only after 12 h. These data further validated the remarkable chiral discrimination between l- and d-Asp–DPPE liposomes in inhibiting Aβ(1–40) fibrillation.

In protein conformational diseases such as AD, Aβ(1–40) peptide featured the conformational transition from a random coil to β-sheet structure, which can be detected by FT-IR^29^ and CD,^30^ as shown in Table S1, Fig. S4 and S5C in the ESI.† Herein, we monitored the influence of l- or d-Asp–DPPE liposomes on the conformational transition of Aβ(1–40) using CD spectroscopy. However, as shown in Fig. S5 in the ESI,† the peaks of chiral liposomes and Aβ(1–40) were overlapped between 190 and 230 nm; after subtracting the signals of the chiral liposomes, the chiral discrimination in the inhibition effect of Aβ(1–40) aggregation was not evident. It is worth noting that the reliability of the CD subtraction process is uncertain, especially for this complex system. A new technique namely microfluidic modulation spectroscopy based on real-time IR detection may be a powerful tool to solve this issue in the future.^31^

It is worth noting that the stability of liposomes was crucial for the repeatability of the experimental results and could not be ignored. Therefore, fluorescence polarization (FP) curves were recorded to reflect the stability of liposomes during their incubation with Aβ(1–40). First, calcein was embedded in l- or d-Asp–DPPE liposomes through freezing–thawing and extrusion operations as previously reported,^32^ obtaining chiral liposomes with uniform size and fluorescent dyes entrapped in the phospholipid bilayers. Under these conditions, free rotation of calcein was restricted, resulting in a high polarization (*P*) value. When the liposome ruptures or converges into a bigger liposome, the entrapped calcein will be released and returns to its freely rotating state, leading to a sharp decrease in the *P* value. Based on this mechanism, the FP of calcein could reflect the fluidity of the phospholipid membrane (Fig. 2D). Then, the calcein-labelled chiral liposomes were incubated with Aβ(1–40) at 37 °C, and the *P* value of calcein was monitored using a multifunctional microplate reader with an FP accessory. As shown in Fig. 2E, during the whole Aβ(1–40) incubation process, no evident change in the *P* value was observed for l- or d-Asp–DPPE liposomes. Combined with the DLS results (Fig. S3 in the ESI†), this was sufficient to prove that these chiral liposomes are stable, providing an ideal biointerface for interaction with Aβ(1–40).

### Morphological observation by AFM

**Fig. 3:**
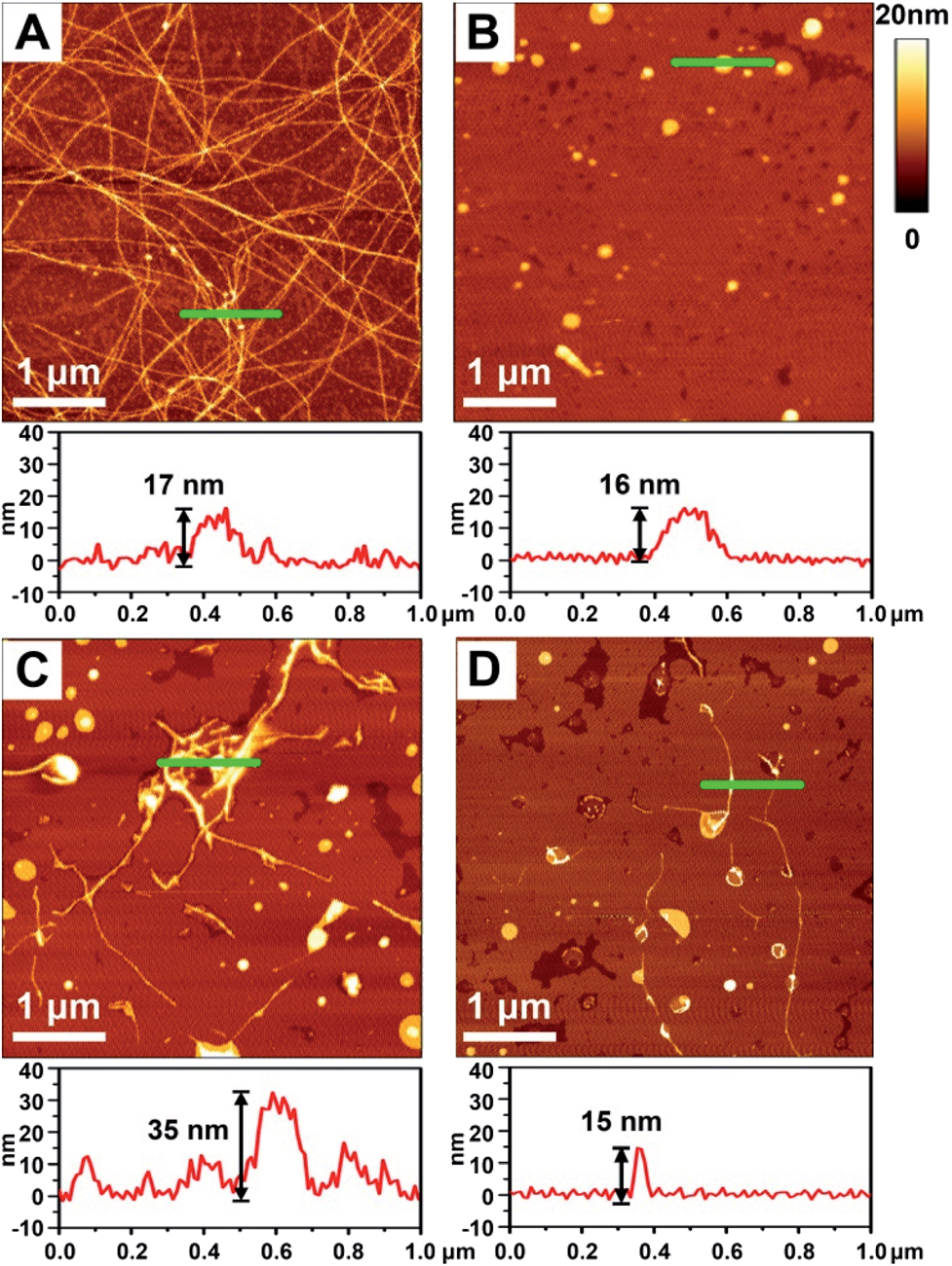
AFM images obtained after incubation of Aβ(1–40) with chiral liposomes at 37 °C for 80 h. (A) Aβ(1–40) alone. (C) Aβ(1–40) with l-Asp–DPPE added. (D) Aβ(1–40) with d-Asp–DPPE added. (B) AFM image of freshly prepared chiral liposomes. The corresponding sectional profile along the green line is shown in the lower panel of each image.

To better demonstrate the chiral discrimination, AFM^33^ was used to observe the morphological changes of Aβ(1–40) incubated with various liposomes. In each experiment, one droplet of peptide solution was dropped onto a freshly cleaved mica surface to prepare the AFM sample. Fig. 3A and S6A in the ESI† show the AFM images of pure Aβ(1–40) after 80 h of incubation at 37 °C. Numerous long fibers were observed across the field of view, and these fibers were intertwined and stacked, resulting in a cumulative height of 17 nm according to the sectional profile of Fig. 3A. Fig. 3B shows the morphology of the prepared liposomes. They were evenly distributed in the field of view and had an average height of 16 nm. As shown in Fig. 3C and D and S6B–D in the ESI,† AFM images of the Aβ(1–40) monomer solution (50 μM) after incubation with various liposomes (1 mg mL^−1^) at 37 °C for 80 h were collected. Regarding the l-Asp–DPPE liposomes, a few short fibers with an average length of 2–3 μm were observed (Fig. 3C and S6C in the ESI†). These short fibers tended to accumulate and intertwine on the liposome surface, as indicated by the white spots, resulting in a remarkable increase of the cumulative height from 17 nm to 35 nm. Interestingly, when d-Asp–DPPE liposomes were studied, the surface was relatively clean. A few short fibers were observed occasionally, and their cumulative height was only 15 nm, as shown in Fig. 3D and S6D in the ESI.† This remarkable difference in the fiber morphology was direct evidence of chiral discrimination.

### Cytotoxicity study

**Fig. 4:**
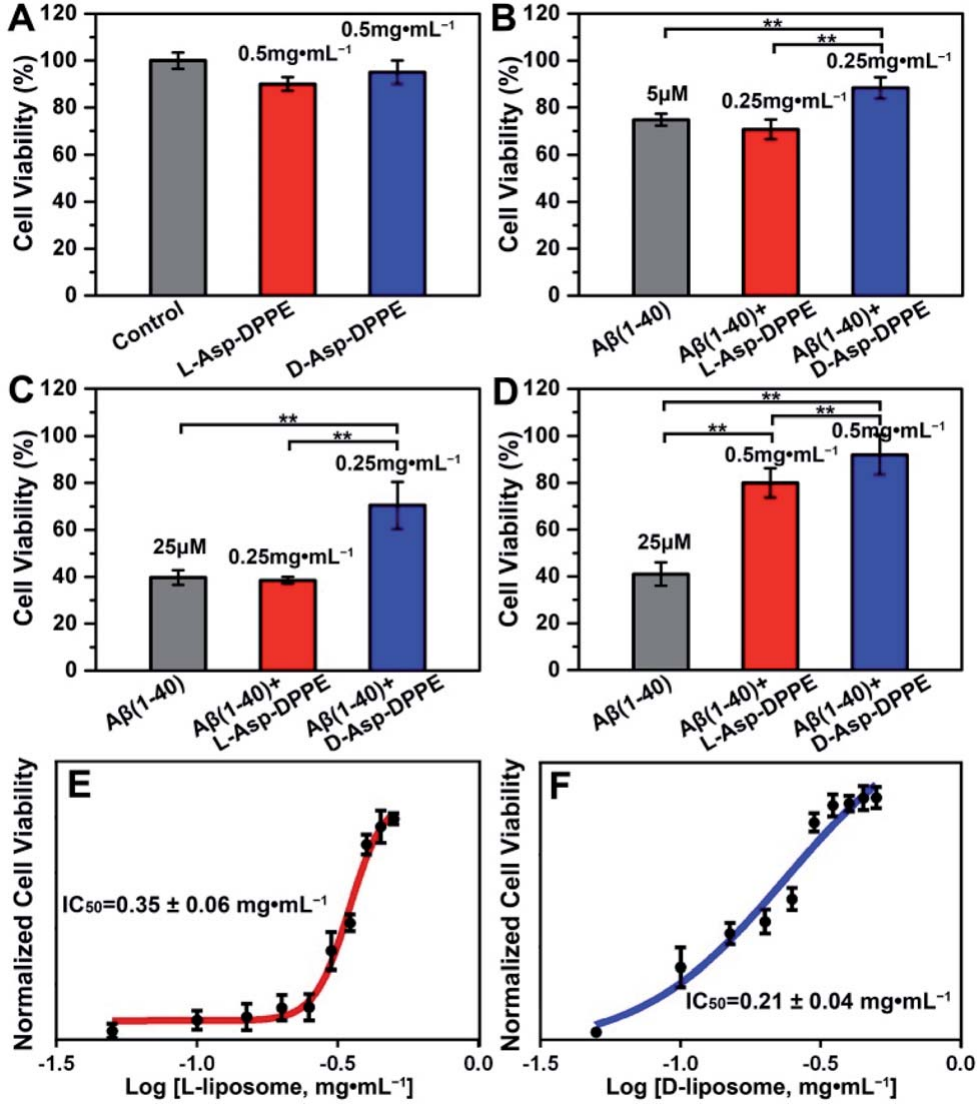
Chiral liposomes rescued the Aβ(1–40)-aggregation induced cytotoxicity in N2a cells. (A) Effects of l- or d-Asp–DPPE liposomes (0.5 mg mL^−1^) on cell viability. (B–D) Effects of l- or d-Asp–DPPE liposomes on Aβ(1–40)-aggregation induced cytotoxicity. The concentrations of Aβ(1–40) are 5 μM (B) and 25 μM (C and D). The concentrations of l- and d-Asp–DPPE liposomes are 0.25 mg mL^−1^ (B and C) and 0.5 mg mL^−1^ (D). (E and F) Dose dependent effect of l- (E) or d-Asp–DPPE liposomes (F) on 25 μM Aβ(1–40) mediated cytotoxicity in N2a cells. Each experiment was repeated three times. Error bars indicate ±s.d. **P < 0.01.

Numerous research studies have revealed that amyloid formation has remarkable cytotoxicity, leading to neuronal cell death by causing structural abnormalities and functional impairments of mitochondria, which plays an important role in the pathogenesis of AD.^34^ In order to evaluate the influence of chiral liposomes on inhibition of cytotoxicity induced by amyloid formation, l- or d-Asp–DPPE liposomes were cocultured with N2a cells (typical mouse neuroblastoma N2a cells) *in vitro* without or with the addition of Aβ(1–40) at different concentrations, and then the cell viability at 72 h was detected by Cell Counting Kit-8 (CCK-8) assay.^35^ Both l- and d-Asp–DPPE liposomes (0.5 mg mL^−1^) displayed satisfying biocompatibility towards the N2a cells, and the cell viability was 90 ± 3% or 95 ± 5%, respectively (Fig. 4A). By comparison, remarkable cytotoxicity was observed when 5 μM or 25 μM Aβ(1–40) was incubated with the N2a cells, and the cell viability sharply decreased to 75 ± 3% or 40 ± 3%, respectively. The addition of d-Asp–DPPE liposomes (0.25 mg mL^−1^) had an obvious inhibition effect on the Aβ(1–40)-aggregation induced cytotoxicity, and the cell viability increased to 88 ± 5% or 70 ± 10%, respectively (Fig. 4B and C). However, under the same conditions, no evident change in the cell viability was observed when l-Asp–DPPE liposomes were added, demonstrating significant chiral discrimination at the cellular scale. Notably, when the concentration of l- or d-Asp–DPPE liposomes was increased to 0.5 mg mL^−1^, the viability of N2a cells exposed to 25 μM Aβ(1–40) reached 80 ± 6% and 92 ± 9%, respectively (Fig. 4D), which indicated that d-Asp–DPPE liposomes had a stronger inhibition effect on the Aβ(1–40)-aggregation induced toxicity than l-Asp–DPPE liposomes.

Evident chiral discrimination was detected by the dose dependent effect of chiral liposomes on Aβ(1–40) mediated cytotoxicity in N2a cells.^35^ In this experiment, to make the experimental conditions consistent with those in the above tests, a concentration of 25 μM was chosen for Aβ(1–40), and the cell viability in the presence of 25 μM Aβ(1–40) and chiral liposomes at the indicated concentrations (from 0.05 mg mL^−1^ to 0.5 mg mL^−1^) was measured. Considering that the l- or d-Asp–DPPE liposome is a self-assembled biomacromolecule that is quite different from small molecule inhibitors, we could only provide two reference IC_50_ values, which were 0.35 ± 0.06 mg mL^−1^ and 0.21 ± 0.04 mg mL^−1^ for l- and d-Asp–DPPE liposomes (Fig. 4E and F and S8 in the ESI†), respectively. Besides, the cytotoxicity of the control experiments of l-Asp, d-Asp and DPPE liposomes at a concentration of 0.5 mg mL^−1^ and their inhibition effect on the 25 μM Aβ(1–40)-aggregation induced toxicity were also evaluated. As shown in Fig. S7 in the ESI,† they had low cytotoxicity but no evident inhibition effect on Aβ(1–40) toxicity. These results highlighted the unique role of the chiral liposomes.

### Adsorption dynamics and binding affinity study

**Fig. 5:**
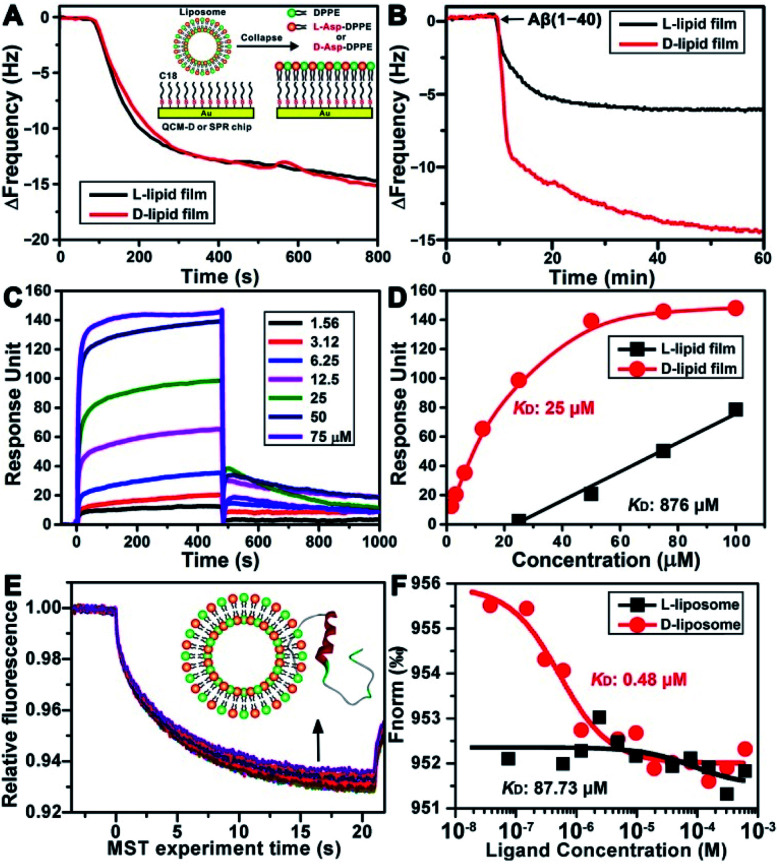
Binding affinity study of Aβ(1–40) with l- or d-Asp–DPPE. (A and B) Dynamic adsorption curves of chiral liposomes adsorbed on octadecyl thiolated gold surfaces (A) and Aβ(1–40) monomers adsorbed on chiral phospholipid surfaces (B), as obtained using QCM. The inset of (A) shows a schematic of a liposome rupturing into a film on the octadecyl thiolated gold surface. (C) Sensorgram of Aβ(1–40) monomers binding to a d-Asp–DPPE-immobilized chip. The Aβ(1–40) concentrations are 1.56, 3.12, 6.25, 12.5, 25, 50, and 75 μM (from the bottom to top). (D) Fitted curves for different concentrations of Aβ(1–40) binding to the l- or d-Asp–DPPE-immobilized surface using an “affinity” model. (E) MST traces of fluorescein-labelled Aβ(1–40) towards d-Asp–DPPE liposomes. The concentrations of d-Asp–DPPE liposomes range from 1 mg mL^−1^ to 3.05 × 10^−5^ mg mL^−1^ (from the bottom to top). The inset of (E) shows a schematic of the interaction of Aβ(1–40) with the liposome. (F) Dose–response curves of fluorescein-labelled Aβ(1–40) towards the addition of l- or d-Asp–DPPE liposomes. The resulting dose–response curves were fitted to a one-site binding model to extract the *K*_D_ values.

To explore the possible mechanism of the chiral effect, a quartz crystal microbalance (QCM) was used to measure the adsorption behavior of Aβ(1–40) on the l- or d-Asp–DPPE phospholipid surface.^36^ In this test, a hydrophobic 1-octadecanethiol monolayer was first assembled on the gold surface of the QCM sensors through thiol–gold bonds. The liposome ruptured into a thin film upon adsorption onto the monolayer (inset in Fig. 5A), which is a typical method for preparing a phospholipid film.^36*b*^ The adsorption dynamics of l- and d-Asp–DPPE liposomes on the QCM sensors were identical (Fig. 5A), both inducing a frequency change (Δ*F*) of 15.0 Hz, corresponding to an adsorption quantity of 88.5 ng cm^−2^ according to the Sauerbrey equation. An Aβ(1–40) peptide solution (25 μM) was then pumped into the cell for evaluating the adsorption capacity of Aβ(1–40) on the chiral phospholipid surface. Aβ(1–40) was discovered to adsorb in a greater amount on the d-Asp–DPPE surface (Δ*F*: 14.4 Hz) than on the l-Asp–DPPE surface (Δ*F*: 6.0 Hz), as shown in Fig. 5B.

Furthermore, surface plasmon resonance (SPR)^37^ was employed to measure the binding affinity of Aβ(1–40) with the l- or d-Asp–DPPE phospholipid surface. The SPR chip was modified with a phospholipid monolayer through the same method as the QCM adsorption experiment. A series of Aβ(1–40) peptide solutions with different concentrations were then pumped into the SPR chip, and the responses were recorded, as illustrated in Fig. 5C. Using a nonlinear fitting to obtain the relationship between response and Aβ(1–40) peptide concentration (Fig. 5D), the equilibrium dissociation constant (*K*_D_) could be obtained, with a smaller *K*_D_ corresponding to stronger binding affinity between the peptide and substrate. The fitting results revealed that the *K*_D_ of Aβ(1–40) with the d-Asp–DPPE surface was 25 μM, substantially smaller than that with the l-Asp–DPPE surface (*K*_D_: 876 μM). This result indicated that the binding affinity of Aβ(1–40) with the d-Asp–DPPE surface was approximately 35 times higher than that with the l-Asp–DPPE surface.

It is worth noting that model phospholipid monolayers used in the QCM adsorption and SPR tests were different from the real liposome environment. To compensate for this deficiency, the interaction between liposomes and Aβ(1–40) was investigated *in situ* by microscale thermophoresis (MST).^38^ As a new technology, MST is based on the thermophoresis of molecules, which allows quantitative analysis of molecular interactions in free solution without surface immobilization, particularly suitable for complex assembly systems, like liposomes and nanomaterials. Fig. 5E shows the MST time traces of fluorescein-labelled Aβ(1–40) towards different concentrations of d-Asp–DPPE liposomes (technical setup diagram of the MST experiment is shown in Fig. S9 in the ESI†). Fitting the data using a one-site binding model resulted in *K*_D_ values. The *K*_D_ of Aβ(1–40) interacting with l- or d-Asp–DPPE liposomes was 87.73 μM or 0.48 μM, respectively (Fig. 5F). A substantially lower *K*_D_ value revealed that Aβ(1–40) had a higher binding affinity for d-Asp–DPPE liposomes than l-Asp–DPPE ones, the tendency of which was consistent with the QCM and SPR results.

### Binding mechanism analysis using the 2D NMR spectra

**Fig. 6:**
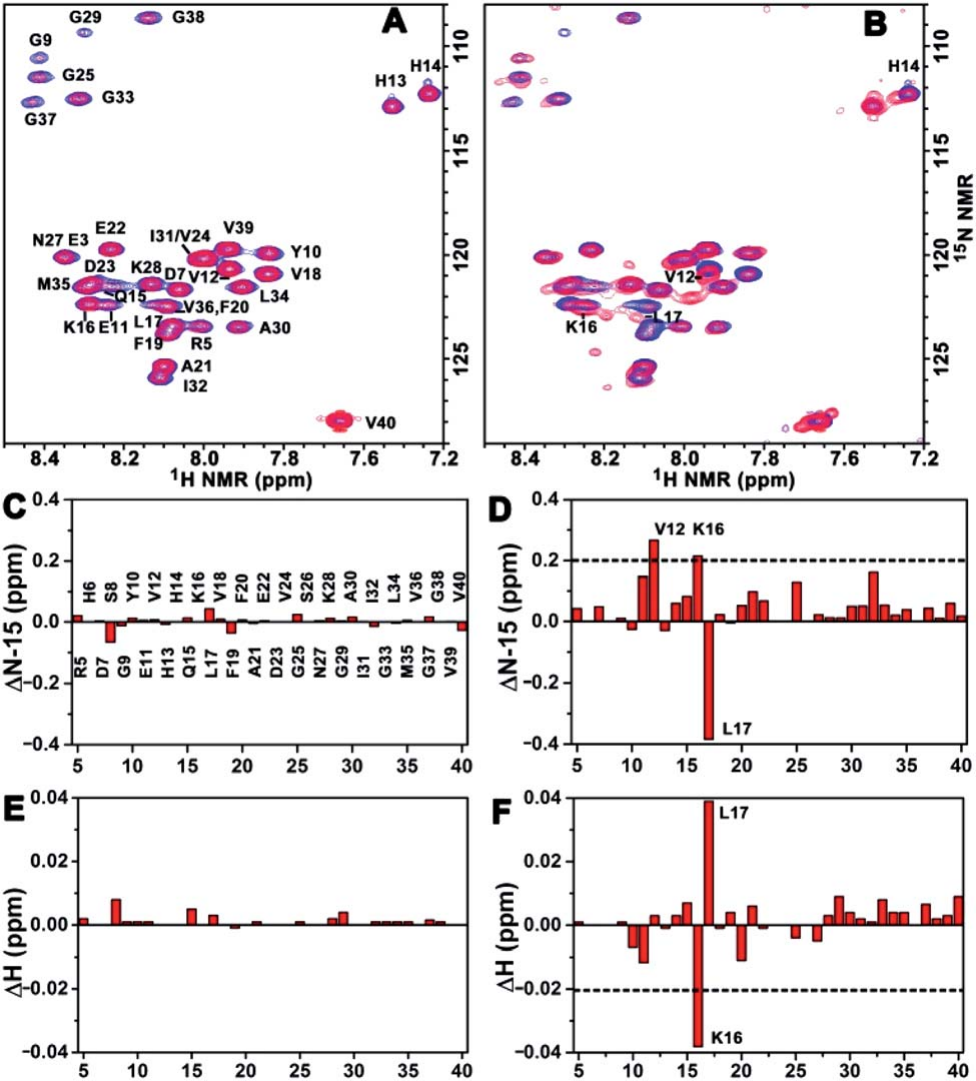
Binding site analysis. (A and B) ^1^H–^15^N HSQC NMR spectra of ^15^N-labelled Aβ(1–40) monomers before (blue peaks) and after (red peaks) interaction with l- (A) or d-Asp–DPPE (B) liposomes. (C–F) NMR chemical shift changes for ^15^N (C and D) or ^1^H (E and F) induced by l- (C and E) or d-Asp–DPPE (D and F) liposomes. The concentrations of Aβ(1–40) and l- or d-Asp–DPPE liposomes are 75 μM and 0.84 mg mL^−1^, respectively. The data were obtained from ^1^H–^15^N HSQC NMR experiments run at 25 °C in a 50 mM phosphate buffer solution containing 10% D_2_O, pH 7.4.

To acquire more detailed information about the molecular interactions between Aβ(1–40) and the chiral liposomes, ^1^H–^15^N HSQC NMR study was carried out. Firstly, the ^1^H–^15^N HSQC NMR spectrum of uniformly ^15^N-labelled Aβ(1–40) (purchased from AlexoTech AB, Sweden) at a concentration of 75 μM was measured at 25 °C, and the peak positions of amide groups on Aβ(1–40) were assigned according to a published assignment.^39^ Secondly, l- or d-Asp–DPPE liposomes were added to an Aβ(1–40) peptide solution, respectively, and the mixtures were measured by NMR under the same conditions. The amide regions of the ^1^H–^15^N NMR spectra were collected as shown in Fig. 6A and B.

Detailed chemical shift changes in the backbone amide groups of Aβ(1–40) induced by the chiral liposomes are presented in Fig. 6C–F. Upon the addition of l-Asp–DPPE liposomes, no evident change in the ^1^H and ^15^N chemical shifts of the amino acid residues of Aβ(1–40) were observed (Fig. 6C and E). By comparison, the addition of d-Asp–DPPE liposomes led to remarkable changes in the chemical shifts (Fig. 6D and F), and the largest effects were detected for residues Lys16 (K16) and Leu17 (L17). Specifically, the nitrogen signals of K16 and L17 shifted from 122.34 to 122.55 ppm and from 123.40 to 123.02 ppm, respectively. Meanwhile, the hydrogen signals of K16 and L17 shifted from 8.29 to 8.25 ppm and from 8.08 to 8.12 ppm, respectively. The sign of the changes in both the ^1^H and the ^15^N dimensions suggested that the molecular interaction of Aβ(1–40) with d-Asp–DPPE liposomes was substantially stronger than that with l-Asp–DPPE liposomes, and the possible binding sites were K16 and L17.

### Binding site validation

Many studies have reported that two key oligopeptide fragments located in the α-helix region of Aβ(1–40), namely Val–His–His–Gln (residues 12–15, abbreviated to VHHQ) and Leu–Val–Phe–Phe (residues 17–20, abbreviated to LVFF), are closely associated with Aβ(1–40) fibrillation.^40^ On the basis of the preceding results, three oligopeptide fragments (*i.e.*, VHHQ, LVFF and VHHQKLVFF) in Aβ(1–40) were selected to investigate their interactions with l- or d-Asp protected by phenylethanamine (abbreviated to PEA l- or d-Asp in the following description), which could be regarded as the chiral head group of l- or d-Asp–DPPE. PEA was chosen to replace DPPE with two hydrophobic alkane chains in order to simplify the interaction model and highlight the role of chiral head groups. Fluorescence (Fig. S10 and S11 in the ESI†) and ^1^H NMR titration experiments (Fig. S12 and S13 in the ESI†) and isothermal titration calorimetry experiments (Fig. S14–S16 in the ESI†) between oligopeptide fragments and PEA l- or d-Asp all demonstrated that the binding capacity of oligopeptides to PEA d-Asp was stronger than that of its enantiomer and also indicated that the electrostatic interactions between the residue K16 in Aβ(1–40) and PEA l- or d-Asp were the source of the chiral effect for the amyloid fibrillation. The detailed experimental description is shown in the ESI. Besides, Brender *et al.*^41^ recently reported that the central domain spanning residues F19–K28 was related to the Aβ(1–40) aggregation pathway, which provided a new direction for future research.

### Molecular dynamics simulation

**Fig. 7:**
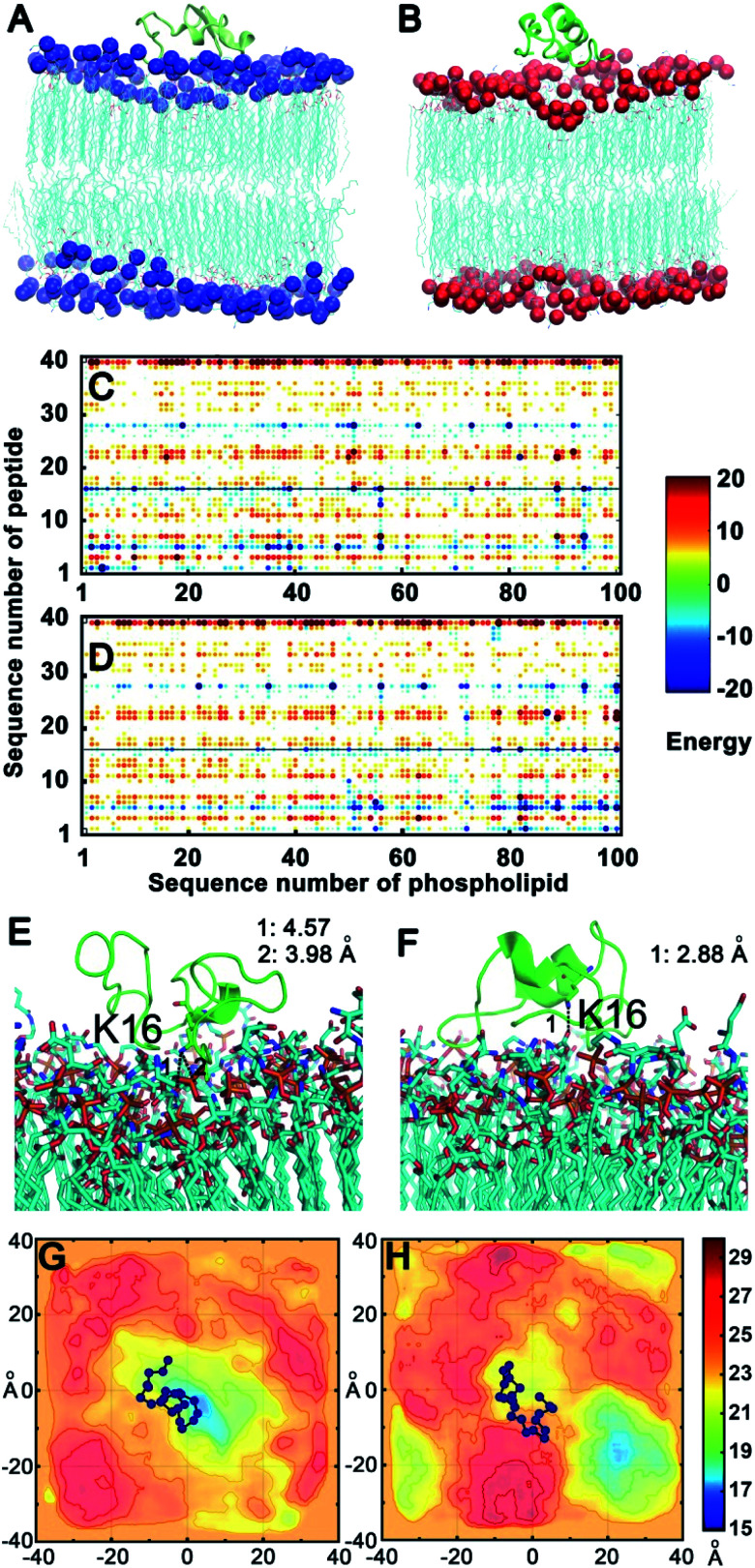
Binding models of Aβ(1–40) with the l- or d-Asp–DPPE phospholipid bilayer. (A and B) Aβ(1–40) binding to the l- (A) or d-Asp–DPPE (B) bilayer; the phosphorus (P) atoms are represented by balls and colored in blue in l-Asp–DPPE and red in d-Asp–DPPE. (C and D) The pairwise interaction energies calculated by the energies between the atoms between Aβ(1–40) and the l- (C) or d-Asp–DPPE (D) membrane model. (E and F) The dominant residue K16, which produces favorable interactions with the two membrane models; Aβ(1–40) is shown as a cartoon, and the dominant residue and membrane models are shown as sticks. (G and H) The mean smooth *P*_z_ surface of the trajectory in the equilibrium state of the l- (G) or d-Asp–DPPE (H) membrane model, and the Aβ(1–40) binding site is shown as balls and lines colored in blue, illustrating that Aβ(1–40) binds to l-Asp–DPPE in the recessed part of the membrane (G), otherwise, the peptide obviously binds to d-Asp–DPPE in the raised part of the membrane (H). The vertical and horizontal axes in (G) and (H) are the scales of the *x*, *y* coordinates of simulation systems. The distribution of the smoothed *P*_z_, after being aligned and shifted, is visualized by continuously varying the colors in the scale from 15 to 30 Å. The color bar represents the smoothed *P*_z_ position, in which the high values represented by dark red indicates the raised part of the lipid and the low values in dark blue indicate the recessed part of the lipid.

To confirm the above experimental results and investigate the binding mode and interaction strength of Aβ(1–40) with the l- or d-Asp–DPPE phospholipid bilayer, the pairwise interaction energies containing vdW and electrostatic interactions were calculated between Aβ(1–40) and l- or d-Asp–DPPE atoms through molecular dynamics simulation;^42^ the detailed calculation method and parameters are displayed in the ESI. The representative structure of each system was selected based on the total interaction energies, and the corresponding structures are shown in Fig. 7A and B. From the analysis of the pairwise interaction energies (Fig. 7C and D), we found that the interaction of Aβ(1–40) with the d-Asp–DPPE bilayer (−75.42 ± 24.30 kcal mol^−1^) was stronger than that with the l-Asp–DPPE bilayer (−51.84 ± 17.26 kcal mol^−1^), while the dominant residues for the interaction energies were R5, K16, and K28 for the two phospholipid models. Through the analysis of the native contact, K16 was found to interact with the head Asp group of the phospholipid intensively through the similar electrostatic interactions in the both models, as shown in Fig. 7E and F. For the d-Asp–DPPE bilayer, the distance between the side-chain amine of the K16 in Aβ(1–40) and the carboxylic acid in d-Asp was 2.88 Å in the representative structure (Fig. 7F), while these values were 3.98 and 4.57 Å for the l-Asp–DPPE bilayer (Fig. 7E), indicating that Aβ(1–40) was closer to the d-Asp–DPPE bilayer than to the l-one.

In addition, analysis of the phosphorus (P) surface distribution (Fig. S17 in the ESI†) showed the different responses of the two phospholipid bilayers to the peptide binding (Fig. 7G and H); in the model of Aβ(1–40) with d-Asp–DPPE, the peptide was bound to the raised part of the phospholipid bilayers, otherwise, the Aβ(1–40) was obviously bound to l-Asp–DPPE in the recessed part of the phospholipid bilayers. The molecular dynamics simulation clearly showed that due to the different chirality of Asp, Aβ(1–40) bound to the membranes in different manners, and the response of the peptide binding to the two phospholipids was distinctly different. Finally, the binding capacity of Aβ(1–40) towards d-Asp–DPPE was stronger than that towards l-Asp–DPPE.

Overall, we presumed that the intensive electrostatic binding between d-Asp on the head of d-Asp–DPPE and the residue K16 in the peptide remarkably promoted the adsorption of Aβ(1–40) monomers on the d-Asp–DPPE phospholipid surface, prevented the random coil to β-sheet transition, and finally inhibited the amyloid fibrillation. By contrast, the binding affinity of the l-Asp–DPPE phospholipid surface with Aβ(1–40) monomers was substantially weaker, the monomers accumulated near the surface, and the conformational transition and fibrillation processes were not influenced strongly.

## Conclusions

In summary, this study described the construction of a pair of chiral liposomes quite similar to the biological membranes and employed them to explore the effect of the chiral interface on amyloid formation. Our study compensates well for the disadvantages of artificial surfaces,^14–19^ independent of the composition and function of the organism itself, which indicates a new direction for chiral interface research. In addition, our results reveal that d-amino acid modified liposomes have a stronger inhibition effect on amyloid fibrillation than the l-ones, which will be a feasible route to design non-natural d-amino acid inhibitors for amyloid formation.^43^

However, this study is only a preliminary exploration and guidance, and more precise structural design and in-depth cell experiments are required to expand and verify this result in future. In particular, liposomes have excellent biocompatibility and can be internalized by cells, which may have great potential for early prevention and treatment of AD when they are combined with various inhibitors,^43,44^ drugs,^45^ or chiral substances.^46^ Besides this, nanoscale liposomes can be regarded as a useful nano-biointerface platform,^47^ which could be specifically modified to have powerful functions such as capturing or identifying DNA, proteins or cells associated with specific diseases.

## Conflicts of interest

There are no conflicts to declare.

## Supplementary Material

SC-011-D0SC02212H-s001
