# Supplementary material for: Molecular chirality mediated amyloid formation on phospholipid surfaces
Source: Chem Sci. 2020 Jun 25;11(28):7369–78. doi: 10.1039/d0sc02212h (PMC8159450; doi:10.1039/d0sc02212h)
Supplement: SC-011-D0SC02212H-s001 [file SC-011-D0SC02212H-s001.pdf]

Electronic Supplementary Material (ESI) for Chemical Science.
This journal is © The Royal Society of Chemistry 2020

Electronic Supplementary Information for

Molecular chirality mediated amyloid formation on phospholipid surfaces

Xue Wang,[ab] Cunli Wang,[b] Huiying Chu,[c] Haijuan Qin,[d] Dongdong Wang,[b] Feifei Xu,[b] Xuanjun Ai,[b] Chunshan Quan,[e] Guohui Li*[c] and Guangyan Qing*[b]

[a]*State Key Laboratory of Advanced Technology for Materials Synthesis and Processing, Wuhan University of Technology, 122 Luoshi Road, Wuhan 430070, P. R. China.*

[b]*Key Laboratory of Separation Science for Analytical Chemistry, Dalian Institute of Chemical Physics, Chinese Academy of Sciences, 457 Zhongshan Road, Dalian 116023, P. R. China.*

[c]*Laboratory of Molecular Modeling and Design, State Key Laboratory of Molecular Reaction Dynamics, Dalian Institute of Chemical Physics, Chinese Academy of Sciences, 457 Zhongshan Road, Dalian 116023, P. R. China.*

[d]*Research Centre of Modern Analytical Technology, Tianjin University of Science and Technology, Tianjin 300457, P. R. China.*

[e]*College of Life Science, Dalian Minzu University, Dalian 116600, P. R. China.*

*Corresponding authors: qinggy@dicp.ac.cn; ghli@dicp.ac.cn

Outline

Materials and Instruments ……………………………………………….…….……….……..… **S3-S4**

Synthesis and Characterization …………………….….…………...……..………. **S4–S6**

Test Methods ………………………………………………...….………...… **S6–S13**

Binding site validation ……………………………………...…………………… **S14**

Figures ……………………………………..……………………….…….. **S15–S32**

References………………………………………………..……..………....... **S32–S33**

Materials and instruments

Materials: Amyloid-β 1−40 (Aβ(1−40), >95%) and fluorescein–labelled amyloid-β 1−40 (fluorescein–Aβ(1−40), >95%) were purchased from Synpeptide Co., Ltd. (China). ^{15}N–labelled amyloid-β 1−40 (^{15}N–labelled Aβ(1−40)) was purchased from AlexoTech AB (Sweden). Oligopeptides (i.e., Val-His-His-Gln-Lys-Leu-Val-Phe-Phe (VHHQKLVFF), Val-His-His-Gln (VHHQ) and Leu-Val-Phe-Phe (LVFF), >95%) and fluorescein–labelled oligopeptides (i.e., fluorescein–VHHQKLVFF, fluorescein–VHHQ and fluorescein–LVFF, >95%) were obtained from ChinaPeptides Co., Ltd. (China). L-Aspartic acid (L-Asp), D-Aspartic acid (D-Asp), Fmoc-Asp(OtBu)-OH, Fmoc-D-Asp(OtBu)-OH, 1-hydroxybenzotriazole (HOBT), 1-Ethyl-3-(3-dimethylaminopropyl)carbodiimide hydrochloride (EDC·I) and 4-dimethyl-amino- pyridine (DMAP) were purchased from GL Biochem. Ind. (China). 1, 2-dipalmitoyl-sn-glycero-3-phosphoethanolamine (DPPE), phenylethylamine (PEA), 1-octadecanethiol, and thioflavin-T (ThT) were purchased from Aladdin Corp. (China). Cell Counting Kit-8 (CCK-8) was purchased from beyotime biotechnology Co., Ltd. (China). N2a cells were obtained from Shanghai (China). High glucose DMEM, 10% FBS, 1% penicillin/streptomycin, and 0.25% trypsin-EDTA were purchased from gibco thermo fisher scientific Co., Ltd. (China). Chloroform, dichloromethane, methanol, ethanol, trifluoroacetic acid (TFA), piperidine, triisopropylsilane (Tis), 1,1,1,3,3,3-hexafluoro-2-propanol (HFIP), sodium hydroxide, sodium phosphate monobasic dihydrate, disodium hydrogen phosphate dodecahydrate, hydrogen peroxide, ammonium hydroxide, dimethyl sulfoxide (DMSO), deuterium oxide (D_2O), deuterated chloroform ($CDCl_3$), deuterated methanol (CD_3OD), and deuterated dimethyl sulfoxide (d_6-DMSO) were used as received. All chemicals used were of chromatographic pure. Water was purified by a Milli-Q system (18.2 MΩ·cm^{-1}).

Instruments: Nuclear magnetic resonance (NMR) spectra were recorded on an AVANCE III HD 700 MHz spectrometer (Bruker, Germany). Mass spectra (MS) were obtained with a Quadrupole Time-of-Flight (Q-TOF) 6540 masshunter (Agilent, USA). Infrared spectra were conducted on an Optics Vertex 80v Fourier transform infrared (FT-IR) spectrometer (Bruker, Germany). X-ray diffraction (XRD) was conducted on a PANalytical X'Pert PRO X-ray (Philips,

Japan) diffractometer with Cu *K*α radiation source (λ= 1.54059 Å). Dynamic light scattering (DLS) measurement was monitored on a Malvern Zetasizer Nano ZS90 (Malvern, UK). Circular dichroism (CD) spectra were recorded on a MOS-450 CD spectrometer (Bio-Logic, France). The absorbance of 450 nm of CCK-8 in cell viability assay was measured on a SpectraMax 2 (MD, America). Quartz Crystal Microbalance (QCM) measurement was conducted on a Q-Sense E4 System (Biolin, Sweden). Atomic Force Microscopy (AFM) investigation was conducted on a NanoWizard Ultra Speed AFM (JPK, Germany) in a QI mode. Surface plasmon resonance (SPR) binding experiment was performed using a Biacore T200 biosensor system (GE Healthcare, Sweden). Microscale thermophoresis (MST) experiment was conducted on a Monolith NT.115 system (Nano Temper Technologies, Germany). Isothermal titration calorimetry (ITC) experiment was conducted on a MicroCal ITC 200 system (Malvern, UK). Fluorescence spectra were recorded on a PerkinElmer FL-6500 Fluorescence Spectrophotometer (PerkinElmer, USA).

Synthesis and Characterization

Synthesis of L-/D-Asp-DPPE and PEA L-/D-Asp

Fmoc-Asp(OtBu)-OH (82 mg, 0.2 mmol) and DPPE (138 mg, 0.2 mmol) were weighted into a dry 100 mL round bottomed flask. EDC·I (96 mg, 0.5 mmol) was added, followed by HOBT (68 mg, 0.5 mmol) and DMAP (61 mg, 0.5 mmol), then chloroform (50 mL). The reaction was heated to 50 °C and stirred overnight. After cooling to room temperature, the solvent was evaporated under reduced pressure and the residue was purified by silica gel column with an elution of dichloromethane and methanol (v/v 25:1). The crude product collected was dissolved in a mixture of 20% piperidine and chloroform (v/v 1:4) and stirred at room temperature for 1 h to cleave the Fmoc protecting group before evaporating the solvents under reduced pressure. Subsequently, TFA, Tis, and H_2O (v/v/v 95:2.5:2.5) were added to the residue and stirred under the same conditions to remove the OtBu protecting group. The solvents were then removed by a gentle stream of nitrogen. After that the crude product was purified again through a silica gel column to obtain the final product as white powder. Similar method was used to synthesize D-Asp-DPPE.

For the synthesis of PEA L-/D-Asp, Fmoc-L- or D-Asp(OtBu)-OH was allowed to react with PEA, other procedures were similar.

Characterization data for L-Asp-DPPE: ^{1}H NMR (400 MHz, $CDCl_3/CD_3OD/D_2O$ (v/v/v:

90:5:5)): δ (ppm): 5.20-5.25 (m, 1H, *C*H*), 4.38-4.43 (m, 2H, OCH_2), 3.91-3.98 (m, 5H, *CH, CH_2, OCH_2), 2.72-2.81 (m, 2H, *CCH_2), 2.25-2.36 (m, 4H, COCH_2), 1.55-1.65 (m, 4H, CCH_2), 1.24-1.30 (m, 50H, CCH_2), 0.85-0.92(m, 6H, CCH_3). ^{13}C NMR (400 MHz, $CDCl_3$/CD_3OD/D_2O (v/v/v: 90:5:5)): δ (ppm): 174.3, 174.0, 70.9, 63.9, 63.0, 34.6, 34.4, 32.3, 30.0, 29.7, 29.5, 25.3, 23.0, 14.1. IR (cm^{-1}): 3429, 2918, 2850, 1741, 1589, 1467, 1243, 1068. MALDI-MS: m/z calcd. for $C_{41}H_{79}N_2O_{11}P$: 806.54; found: 807.54 [M+H]$^+$. Elemental analysis calcd. (%) for $C_{41}H_{79}N_2O_{11}P$: C, 61.02; H, 9.87; N, 3.47; P, 3.84. Found: C, 60.92; H, 9.75; N, 3.58; P, 3.62.

Characterization data for D-Asp-DPPE: ^{1}H NMR (400 MHz, $CDCl_3$/CD_3OD/D_2O (v/v/v: 90:5:5)): δ (ppm): 5.20-5.25 (m, 1H, *C*H*), 4.38-4.43 (m, 2H, OCH_2), 3.91-3.98 (m, 5H, *CH, CH_2, OCH_2), 2.72-2.81 (m, 2H, *CCH_2), 2.25-2.36 (m, 4H, COCH_2), 1.55-1.65 (m, 4H, CCH_2), 1.24-1.30 (m, 50H, CCH_2), 0.85-0.92(m, 6H, CCH_3). ^{13}C NMR (400 MHz, $CDCl_3$/CD_3OD/D_2O (v/v/v: 90:5:5)): δ (ppm): 174.3, 174.0, 70.9, 63.9, 63.0, 34.6, 34.4, 32.3, 30.0, 29.7, 29.5, 25.3, 23.0, 14.1. IR (cm^{-1}): 3429, 2918, 2850, 1741, 1589, 1467, 1243, 1068. MALDI-MS: m/z calcd. for $C_{41}H_{79}N_2O_{11}P$: 806.54; found: 807.54 [M+H]$^+$. Elemental analysis calcd. (%) for $C_{41}H_{79}N_2O_{11}P$: C, 61.02; H, 9.87; N, 3.47; P, 3.84. Found: C, 60.87; H, 9.79; N, 3.63; P, 3.57.

Characterization data for PEA L-Asp: ^{1}H NMR (400 MHz, d_6-DMSO): δ (ppm): 8.46 (t, *J*=6 Hz, 1H, CON*H*), 8.14 (br, 2H, NH_2), 7.29-7.32 (m, 2H, Ar-*H*), 7.21-7.23 (m, 3H, Ar-*H*), 3.96-3.99 (m, 1H, **H*), 3.39-3.46 (m, 2H, NCH_2), 2.67-2.75 (m, 4H, CCH_2). ^{13}C NMR (400 MHz, d_6-DMSO): δ (ppm): 171.5, 168.0, 139.5, 129.1, 128.9, 126.7, 49.5, 36.0, 35.3, 14.44. IR (cm^{-1}): 3328, 3215, 1668, 1297, 1188, 1137, 834, 798, 751, 723, 704. MALDI-MS: m/z calcd. for $C_{12}H_{16}N_2O_3$: 236.12; found: 237.12 [M+H]$^+$. Elemental analysis calcd. (%) for $C_{12}H_{16}N_2O_3$: C, 61.00; H, 6.83; N, 11.86. Found: C, 60.86; H, 6.72; N, 11.97.

Characterization data for PEA D-Asp: ^{1}H NMR (400 MHz, d_6-DMSO): δ (ppm): 8.43 (t, *J*=6 Hz, 1H, CON*H*), 8.13 (br, 2H, NH_2), 7.28-7.32 (m, 2H, Ar-*H*), 7.21-7.23 (m, 3H, Ar-*H*), 3.96-3.99 (m, 1H, **H*), 3.39-3.46 (m, 2H, NCH_2), 2.67-2.75 (m, 4H, CCH_2). ^{13}C NMR (400 MHz, d_6-DMSO): δ (ppm): 171.5, 168.0, 139.5, 129.1, 128.9, 126.7, 49.5, 36.0, 35.3, 14.44. IR (cm^{-1}): 3328, 3215, 1668, 1297, 1188, 1137, 834, 798, 751, 723, 704. MALDI-MS: m/z calcd. for $C_{12}H_{16}N_2O_3$: 236.12; found: 237.12 [M+H]$^+$. Elemental analysis calcd. (%) for $C_{12}H_{16}N_2O_3$: C, 61.00; H, 6.83; N, 11.86. Found: C, 60.90; H, 6.77; N, 11.99.

Preparation of peptide and liposomes

A typical method was used to prepare Aβ(1−40) peptide solution.[1] Aβ(1−40) peptide powder was first dissolved in HFIP at a concentration of 1 mg·mL^{-1}. It was vortexed at room temperature for 2 h at moderate speed. The stock solution was then dried under a gentle stream of nitrogen to remove HFIP. The peptide was dissolved in 100 μL DMSO and stored at −20 °C. Prior to use, the peptide in DMSO was diluted with phosphate buffer solution (PBS) (50 mM, pH 7.4) to the desired concentration. The addition of DMSO was to improve the solubility of the peptide.[2–4]

Liposomes were obtained through a classical extrusion method.[5,6] L-Asp-DPPE (2 mg) mixed with DPPE (2 mg) was dissolved in a mixture of chloroform and methanol (v/v 2:1). Evaporation of the solvent under reduced pressure causes the residue to form a film at the bottom of the flask. To prepare liposomes, the dried lipid film was allowed to rehydrate by adding 4 mL of PBS and heated to 70 °C under stirring for at least 1 h. The multilamellar vesicle suspension was then extruded through a mini-extruder (Avanti Polar Lipids Inc.). A polycarbonate membrane with 0.1 μm pore diameter was used and nineteen extrusion cycles were performed. The resulting clear unilamellar vesicle solution at a concentration of 1 mg·mL^{-1} was stored in a vial and used in fresh. Similar method was used to prepare other unilamellar vesicles. Unless otherwise stated, the chiral liposomes used in this work (i.e., L-Asp-DPPE and D-Asp-DPPE) were prepared by mixing L- or D-Asp-DPPE and DPPE at a mass ratio of 1: 1.

Test methods

ThT fluorescence spectroscopy experiment

To study the influence of various liposomes on nucleation phase of Aβ(1−40) peptide, a series of working solutions (100 μL) were prepared containing 50 μM Aβ(1−40) peptide, 50 μM ThT in PBS. One of various liposomes (i.e., L- and D-Asp-DPPE, DPPE) or amino acid small molecules (i.e., L- and D-Asp) (100 μL, 1 mg·mL^{-1}) was then added to the pre-prepared solutions. The liposome was replaced by PBS in the blank experiment. Dynamic growth curves were monitored by a standard ThT fluorescence assay. Fluorescence data were record using a Synergy™ H1M Multifunctional Microplate Tester with a bottom-reading mode in 96-well flat bottom plates sealed with a platemax film. Plates were shaken for 2 s before reading fluorescent data every 10 min using an excitation wavelength of 445 nm and emission wavelength of 485 nm at 37 °C. Each

experiment was run in triplicated in a 96-well plate.

To study the influence of the chiral liposomes on the fiber elongation phase, the mixtures of 50 μM Aβ(1−40) peptide and 50 μM ThT in PBS were incubated in a 37 °C water bath until the Aβ(1−40) has become oligomers. After that, the peptide solutions (100 μL) were diluted by 100 μL solution of chiral liposomes (1 mg·mL^{-1}). Subsequently, dynamic fibrillation processes were monitored by ThT fluorescence assay as described above. 1% DMSO was used in the ThT binding assays.

Circular dichroism (CD) spectroscopy experiment

L-/D-Asp-DPPE and DPPE powders were each dissolved in a mixture of dichloromethane and methanol (v/v 4:1) at a concentration of 2 mg·mL^{-1}. L- and D-Asp aqueous solution (1 mg·mL^{-1}) and newly extruded L- and D-Asp-DPPE liposomes (1 mg·mL^{-1}) were also prepared. CD spectra were recorded in a range of 220 to 280 nm or 190 nm to 260 nm at room temperature.

HFIP pre-treated Aβ(1−40) was first dissolved in 100 μL NaOH aqueous solution (1 mM), then diluted by PBS. The work solutions containing 300 μL of Aβ(1−40) solution (50 μM) and equivalent volume of L- or D-Asp-DPPE liposomes (1 mg·mL^{-1}) were incubated in a 37 °C water bath. The sample was then transferred into a quartz cuvette (light path: 1 mm, volume: 1 mL) for CD test. CD spectra were recorded every 24 h in a range of 190 to 250 nm at room temperature. And the CD spectra of Aβ(1−40) solution (25 μM) during fibrillogenesis at 37 ºC were measured used the same method. Raw data were manipulated by smoothing and subtracting the buffer spectrum. As for the samples of Aβ(1−40) with the additions of chiral liposomes, the final curves were subtracted the spectrum of the corresponding chiral liposomes.

Fourier transform infrared (FT-IR) spectroscopy

Infrared spectra of phospholipid molecules (i.e. L-Asp-DPPE, D-Asp-DPPE) were recorded on a Bruker Vertex 80v FT-IR spectrometer with a Bio-ATR accessory. Each sample powder was tested at 20 °C. The parameters were 4 cm^{-1} for the spectral resolution, 4000 to 400 cm^{-1} for the measurement range.

Infrared spectrum of Aβ(1−40) was recorded on a Thermofisher scientific Nicolet iS50 FT-IR spectrometer in an ATR mode (Thermofisher, USA). First, 25 μM Aβ(1−40) solution was prepared (identical to the CD experiment) and lyophilized. Then, the sample powder was tested at

20 °C. The parameters were 4 cm^{-1} for the spectral resolution, 4000 to 400 cm^{-1} for the measurement range. Final, the software of OMNIC and XPSPEAK41 were used for data processing. OMNIC software was used to obtain a deconvolution spectrum and a second derivative spectrum of the amide I absorption band (1700–1600 cm^{-1}) of Aβ(1−40) in order to find the position of each peak. XPSPEAK41 software was used to perform curve fitting until the synthetic curve matched the experimental one. The proportion of the secondary structures was computed to be the fractional area of the corresponding peak, divided by the sum of the areas of all the peaks. The β-sheet (1612–1640 cm^{-1}), α-helix (1650–1657 cm^{-1}), random coil (1640–1651 cm^{-1}) and β-turn (1655–1675 cm^{-1} and/or 1680–1696 cm^{-1}) structures are determined on the basis of previous reports.[7,8]

Fluorescence polarization experiment

Calcein–loaded chiral liposomes were prepared as described above. In brief, chiral phospholipid solutions were first prepared in a mixture of chloroform and methanol (v/v 2:1) and dried in round-bottom flasks by rotary evaporation under reduced pressure. The obtained homogeneous films were then rehydrated in a PBS solution containing 100 mM calcein to form multilamellar vesicles. After five cycles of freezing-thawing treatment, the free calcein was removed by centrifugation at 12000 rpm for 15 min and washed with PBS. Then the chiral liposome was dispersed in PBS at a concentration of 1 mg·mL^{-1}. Finally, the chiral liposome size was adjusted by the extrusion of liposome suspension through a polycarbonate membrane with 0.1 μm pore diameter.

A series of working solutions (200 μL) containing 100 μL calcein–loaded chiral liposomes (i.e., L- and D-Asp-DPPE) (1 mg·mL^{-1}) and 100 μL Aβ(1−40) peptide (50 μM) were prepared similarly to the ThT fluorescence spectroscopy experiment. In the blank experiment, the Aβ(1−40) peptide was replaced by PBS. The polarization (*P*) value was recorded in the fluorescence polarization mode of the Synergy™ H1M Multifunctional Microplate Tester. Except for the excitation and emission wavelengths changed to 485 nm and 528 nm, respectively, other parameters were consistent with the ThT fluorescence spectroscopy experiment. 1% DMSO was used in this experiment. The *P* value was calculated by the following equation:

$$P = \frac{I_{vv} - GI_{vh}}{I_{vv} + GI_{vh}}$$

where I_{vv} is the intensity of fluorescence parallel to excitation plane, I_{vh} is the intensity of fluorescence perpendicular to excitation plane, G is the grating correction coefficient, $G = I_{hv}/I_{hh}$.

Dynamic light scattering experiment

A Zetasizer Nano system was employed with an argon-neon laser (λ= 633 nm, θ= 173°). Newly extruded liposomes (i.e., L-and D-Asp-DPPE, DPPE) in PBS of 1 mg·mL^{-1} or samples (Aβ(1−40) alone, Aβ(1−40) with L- or D-Asp-DPPE liposomes added) incubated at 37 °C for 80 h were placed in a quartz cuvette (light path: 10 mm, volume: 3.5 mL). After waiting for 120 s (well dispersed and stabilized), the mean hydrodynamic diameter was obtained by an average of 12 measurements at 25 °C, and each data was collected in 10 s intervals. Due to the measure range of DLS is 2 nm−3 μm, the measured data beyond the range are for reference only. The preparation method of peptide solutions was the same as the ThT binding assay, 1% DMSO was used.

Atomic force microscopy experiment

AFM measurements were conducted on a NanoWizard Ultra Speed AFM (JPK, Germany) in a QI mode. The samples after ThT binding asssay were used for AFM study. Sample was prepared by dropping 10 μL of peptide solution or chiral liposomes on a freshly cleaved mica and allowed it to dry in the air. Then AFM images were acquired under ambient conditions.

Cell viability assay

Cell viability was determined using the Cell Counting Kit-8 (CCK-8) assay in N2a cells. N2a cells were cultured in high glucose DMEM, supplemented with 10% FBS and 1% penicillin/streptomycin, in 5% CO_2 at 37 °C. Once the cells reached 95% confluence, they were split (using 0.25% trypsin-EDTA), seeded at a density of 5 × 10^3 cells/well in 100 μL complete medium in 96-well plates and cultured for 48 h. After that, the medium was removed, the cells were washed with PBS and 100 μL serum-free DMEM was added to the wells. To investigate the chiral liposomes rescued Aβ(1−40)–aggregation induced cytotoxicity in N2a cells, each well contained 100 μL cells, 50 μL Aβ(1−40) and 50 μL L or D-Asp-DPPE liposomes. Meanwhile, the toxic effect of chiral liposomes alone, and Aβ(1−40) alone on cell viability were determined. After culturing for 72 h in 5% CO_2 at 37 °C, CCK-8 reagent was added to each well and incubated for 2 h at 37 °C. Then the absorbance of 450 nm of CCK-8 was measured on a SpectraMax 2, with a reference wavelength of 600 nm to subtract the background. Wells treated with a solution

containing PBS served as control. CCK-8 reduction was determined from the ratio of the absorbance of the treated wells to the control wells. The control experiments of L-Asp, D-Asp, and DPPE liposomes were evaluated in the same method as above. Each data point represents an average of three independent quintuplet-well trials. Considering the cell tolerance, 0.5% DMSO was added in the cell viability assay.

Adsorption experiment of Aβ(1−40) peptide

The gold coated resonators (frequency: 4.95 MHz ± 50 kHz, diameter: 14 mm) used for adsorption monitored by QCM were cleaned according to the standard protocol: the resonators were washed in a mixed solution of H_2O, $NH_3 \cdot H_2O$, and H_2O_2 (v/v/v 5:1:1) at 75 °C for 15 min. The clean resonators were rinsed by water and dried under nitrogen, then were immersed in an ethanol solution of 1-octadecanethiol (1 mM) for at least 12 h to form a hydrophobic monolayer on gold surface by thiol−gold bonds. Subsequently, the chemically modified resonators were washed with plenty of water and dried under nitrogen. The QCM channels and tubes were washed with water carefully. Prior to binding assays between chiral liposomes and Aβ(1−40), liposomes (1 $mg \cdot mL^{-1}$) were injected into the channels to obtain self-assembled monolayers. Then, Aβ(1−40) peptide solution (25 μM) containing 1% DMSO was pumped into the channels. All the QCM measurements were taken at 25 °C at a flow rate of 0.1 $mL \cdot min^{-1}$, and dynamic adsorption curves were recorded by Q-Sense software and analyzed by Q-Tools.

Binding affinity measurement using SPR and MST.

Biacore T200 and sensor chip gold were used to measure the interaction between chiral liposomes and Aβ(1−40) monomers. All the SPR−based materials were acquired from GE Healthcare. Prior to testing, the sensor chip gold was modified as described in the QCM experiment. L- or D-Asp-DPPE liposomes (1 $mg \cdot mL^{-1}$) were immobilized on a octadecanethiol−modified sensor chip gold and formed phospholipid monolayers. A total of 700 RU of the immobilized liposomes was obtained. A series of Aβ(1−40) monomers solutions (1.56, 3.12, 6.25, 12.5, 25, 50, and 75 μM) containing 1% DMSO were prepared by running PBS. Sensor preparation and interaction analyses were performed at 25 °C. Reference flow cell without immobilized liposomes served as a non-special binding control. Biacore traces were baseline subtracted and the signal was presented in sensorgrams and measured in RU. Equilibrium

dissociation constants (K_D) were calculated using the "Affinity" model in Biacore T200 evaluation software version 2.0.

MST experiments were conducted on a Monolith NT.115 system. The fluorescein–labelled Aβ(1−40) peptide solution with a concentration of 200 nM containing 0.009% DMSO was prepared in 10 mM PBS (pH 7.4) containing 0.05% Tween-20. A two-fold dilution series of unlabelled L- and D-Asp-DPPE liposomes were prepared in the same buffer, with the concentration ranging from 1 mg·mL^{-1} to 3.05×10^{-5} mg·mL^{-1}. Then, fluorescein–labelled Aβ(1−40) peptide solution and L- or D-Asp-DPPE liposomes were thoroughly mixed at a volume ratio of 1:1. After that, samples were drawn into premium coated capillaries and subjected to MST analysis at 25 °C. The K_D values were determined using a one-site model to fit the raw trace curves.

2D NMR spectroscopy

2D NMR experiments were performed on a Bruker AVAVCE III HD 700 MHz spectrometer. ^{15}N–labelled Aβ(1−40) was prepared in D_2O:H_2O (v/v 1:9) according to a previously published protocol with a slight modification.[6] Briefly, the peptide was dissolved in NaOH (1 mM) yielding a concentration of 2 mg·mL^{-1} and sonicated in an ice bath for 1 min. PBS (50 mM, pD 7.4) was added to obtain monomeric samples at a concentration of 75 μM. Chiral liposomes (i.e., L-Asp-DPPE and D-Asp-DPPE) were also prepared using PBS (50 mM, pD 7.4) at a concentration of 1 mg·mL^{-1}. The final concentrations of peptide and chiral liposomes were 75 μM and 0.84 mg·mL^{-1}, respectively. 2D NMR spectra were recorded at 25 °C immediately when the samples were prepared. The ^{1}H–^{15}N HSQC spectrum of Aβ(1−40) was assigned according to a published assignment.[10]

Fluorescence titration experiment

Fluorescence titration experiments were carried out in a PerkinElmer FL-6500 fluorescence spectrometer with a 10 mm path-length quartz cuvette. Host fluorescein–labelled oligopeptides (i.e., VHHQKLVFF, VHHQ, and LVFF) were prepared as stock solutions in H_2O containing 0.2% DMSO for 5.0×10^{-6} mol·L^{-1}. Guest PEA L- and D-Asp were prepared to 1.5×10^{-2} mol·L^{-1}, 1.5×10^{-3} mol·L^{-1}, and 1.5×10^{-4} mol·L^{-1} of stock solution in H_2O containing 0.2% DMSO, respectively. The mother liquor of 3 mL of fluorescein–labelled oligopeptides solution was titrated

by continuous addition of the guest solution. The fluorescence intensity of the work solutions was measured at 25 °C. Association constant (K_a) values were obtained from fluorescence titration experiments according to intensity changes in the emission-peak maximum by nonlinear fitting.

^{1}H NMR titration experiment

To validate the combination between oligopeptides (i.e., VHHQ, LVFF) and PEA L- and D-Asp, ^{1}H NMR titration experiments were performed to investigate the binding details in d_6-DMSO at 20 °C. Different molar amounts (0−2 equiv.) of PEA L- and D-Asp (0.02 mol·L^{-1}) were added to the host solution of oligopeptides solution (0.02 mol·L^{-1}) and then the chemical shift changes of active hydrogen protons were recorded and analyzed.

Isothermal titration calorimetry experiment

The isothermal titration was performed by Microcal ITC 200 with a 200 μL cell and a 40 μL syringe. All the experiments were conducted at 25 ºC, while the stir speed and time interval were set to 750 rpm and 100 s, respectively. The injection number was 20 and the injection volume for 2-20th injection was 2 μL, whereas the first injection volume was set as 0.4 μL. Both VHHQKLVFF (1 mM) and PEA L-/D-Asp (10 mM) were dissolved in H_2O containing 5% DMSO sufficiently, in which PEA L-/D-Asp was titrant and VHHQKLVFF was titrand. The control experiment was conducted under the same conditions, using H_2O containing 5% DMSO as titrant and the titrand. Besides, both VHHQKLVFF (1 mM) and L-/D-Asp (10 mM) were dissolved in H_2O, and L-/D-Asp was titrated to VHHQKLVFF was conducted under the same conditions. The reaction heat was recorded during the binding process, and subtracting the control experiment using software gave the corrected reaction heat for each injection. Then the data was fitted using a one-site model.

Molecular dynamics simulation

To mimic the experimental condition, two model membranes were composed of 200 L- or D-Asp-DPPE and DPPE phospholipids with a mixture ratio of 1:1. Then the systems were solvated with TIP3P water molecules. The monomer of Aβ(1−40) (PDB code 2LFM)[11] was used to construct the model of peptide binding to model membrane. Ten models of Aβ(1−40) binding to two heterogeneous bilayers were built. The charges of the systems were balanced to neutral using 150 mM NaCl, respectively. The electronic potential of the head of L- and D-Asp-DPPE was

calculated using the Gaussian 09 program with the B3LYP functional under 6-311G* basis set.[12] The partial charges of the substrate molecules were derived using the RESP charge fitted with the antechamber module in Amber 16. The other parameters, including vdW, bond, angle and torsion terms, were obtained with the antechamber module.[13] All molecular dynamics (MD) simulations were performed using Amber 16 package[13] under NPT condition. The leap-frog integrator[14] was used with an integration time-step of 2 fs. The calculation of electrostatic interactions was performed using the Particle-Mesh Ewald algorithm[15] with a cutoff of 12 Å. The same cutoff value was chosen for treating the van der Waals interactions.

The semi isotropic pressure coupling was employed using the Monte Carlo barostat[16] controlled the pressure at 1 bar with a coupling constant of 5 ps when the production run was performed. To maintain the stability of the lipid system, all simulations were performed above the experimental liquid-crystalline phase transition temperature (~315 K for pure dipalmitoyllecithin).[17] The Langevin thermostat[18] was employed to couple the temperature of the systems around 323.5 K[19] with a time constant of 1 ps. For each system, three independent MD simulations have been carried out and each simulation was performed for 1 μs, and totally run for 60 μs. The process of protein binding to membrane needs more simulation time to equilibrium, the last 500 ns of each simulation were used to analysis, and the analysis of interaction energies, native contacts etc. were performed by cpptraj module in Amber 16.[20]

The positions of P atom of phospholipid molecules were statically analyzed to investigate the difference of L- and D-Asp-DPPE behaviors, when the Aβ(1−40) binding to it. The phospholipid bilayers were aligned and shifted to make the membrane molecules geometrical center on the origin of coordinate. All the phospholipid molecules were chosen for statically analysis. The *z* coordinates of P atom were applied to fit the P_z surface. And the median filtering[21] was applied to smooth the surface. 500 frames after the system reached the equilibrium state were analyzed according to the steps mentioned above. And the mean z surface of the *P* surface distribution was got by following formula:

$$P_z = \frac{\sum_{i=1}^{n_{fr}} P_i}{n_{fr}}$$

where P_z is the mean of P_i. P_i is the P_z surface in ith frame of MD trajectory of equilibrium state.

n_{fr} is the total number of the frame applied in static process. The illustration of fitting the P_z surface of the equilibrium trajectory was shown in Fig. S17.

Binding site validation

First, fluorescence titration experiments were performed to calculate association constant (K_a) according to remarkable fluorescence quenching of fluorescein−labelled oligopeptide caused by the addition of PEA L- or D-Asp (Fig. S10 in ESI). The calculation results revealed that the K_a of VHHQKLVFF with PEA D-Asp was 89920 L•mol^{-1}, which was larger than that with PEA L-Asp (K_a: 56684 L•mol^{-1}), validating the chiral discrimination observed by the $^1H-^{15}N$ HSQC NMR spectra. Remarkable differences between PEA L- and D-Asp were also detected when they interacted with VHHQ or LVFF, observed by the fluorescence (Fig. S10 in ESI) and ^1H NMR titration experiments (Fig. S12 and S13 in ESI). However, their K_a values only ranged from 968 to 5344 L•mol^{-1}, which were substantially smaller than that with VHHQKLVFF (Fig. S11 in ESI). These results indicated that the electrostatic interactions between the residue K16 in Aβ(1−40) and PEA L- or D-Asp were the source of the chiral effect for the amyloid fibrillation.

The binding preference of VHHQKLVFF for PEA D-Asp was further confirmed by isothermal titration calorimetry (ITC), which is a powerful technique to detect the thermodynamic parameters of interaction between molecules without label.[22] With the titration of PEA D-Asp in VHHQKLVFF solution, endothermic data were recorded, and the optimal fitting curve yielded a K_a value of 3590 L•mol^{-1} (Fig. S14A and S14C in ESI). Under the same condition, when PEA L-Asp was added to VHHQKLVFF solution, the endathermic data were too small to give a reliable K_a value (Fig. S14B and S14D in ESI). Therefore, the binding affinity results indicated the stronger binding capacity of VHHQKLVFF towards PEA D-Asp than its enantiomer.

Figures

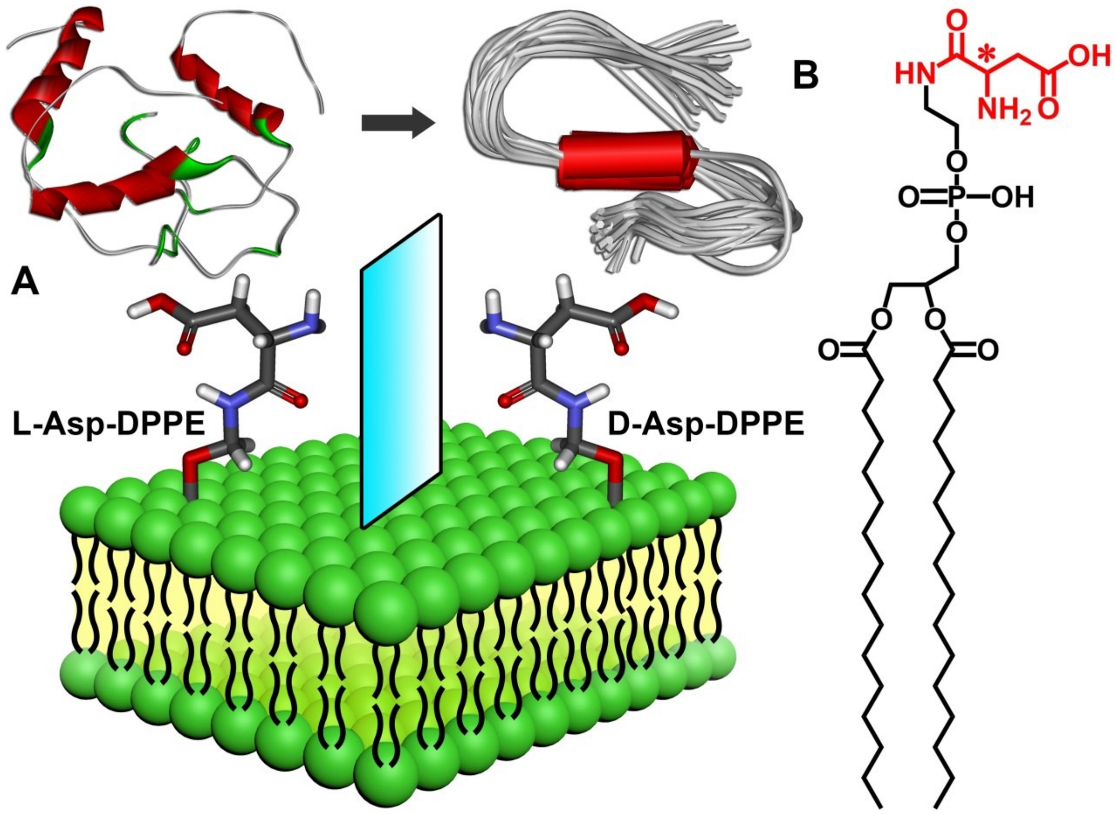

Scheme S1 Schematic of the interaction of chiral phospholipid surface with amyloid-β. (A) Effect on amyloid formation of the molecular chirality of an Asp enantiomer–modified phospholipid bilayer. (B) Chemical structure of L- or D-Asp–modified DPPE studied in this work, abbreviated to L- or D-Asp-DPPE.

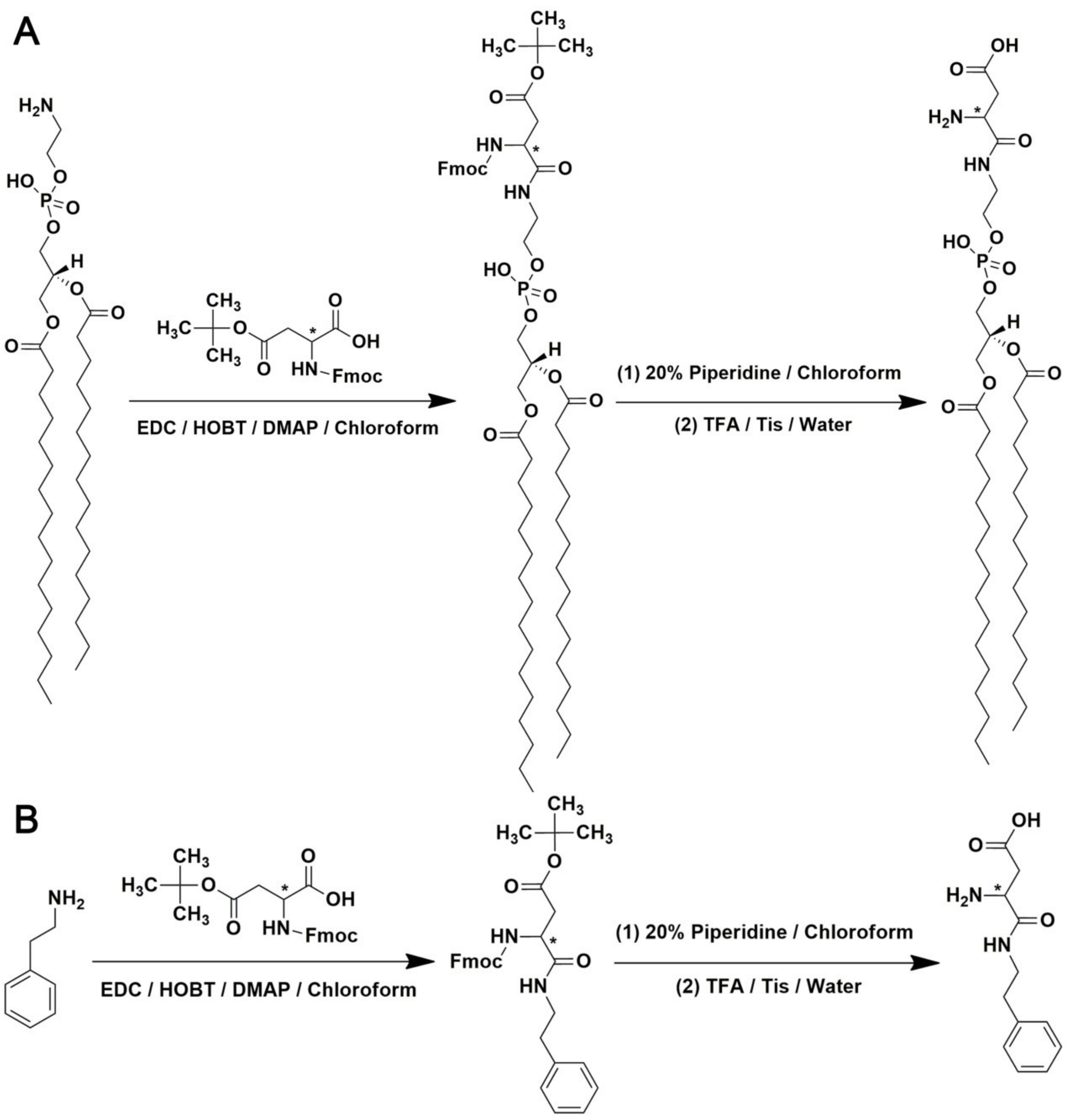

Scheme S2 Synthesis route of L-/D-Asp-DPPE (A) and PEA L-/D-Asp (B).

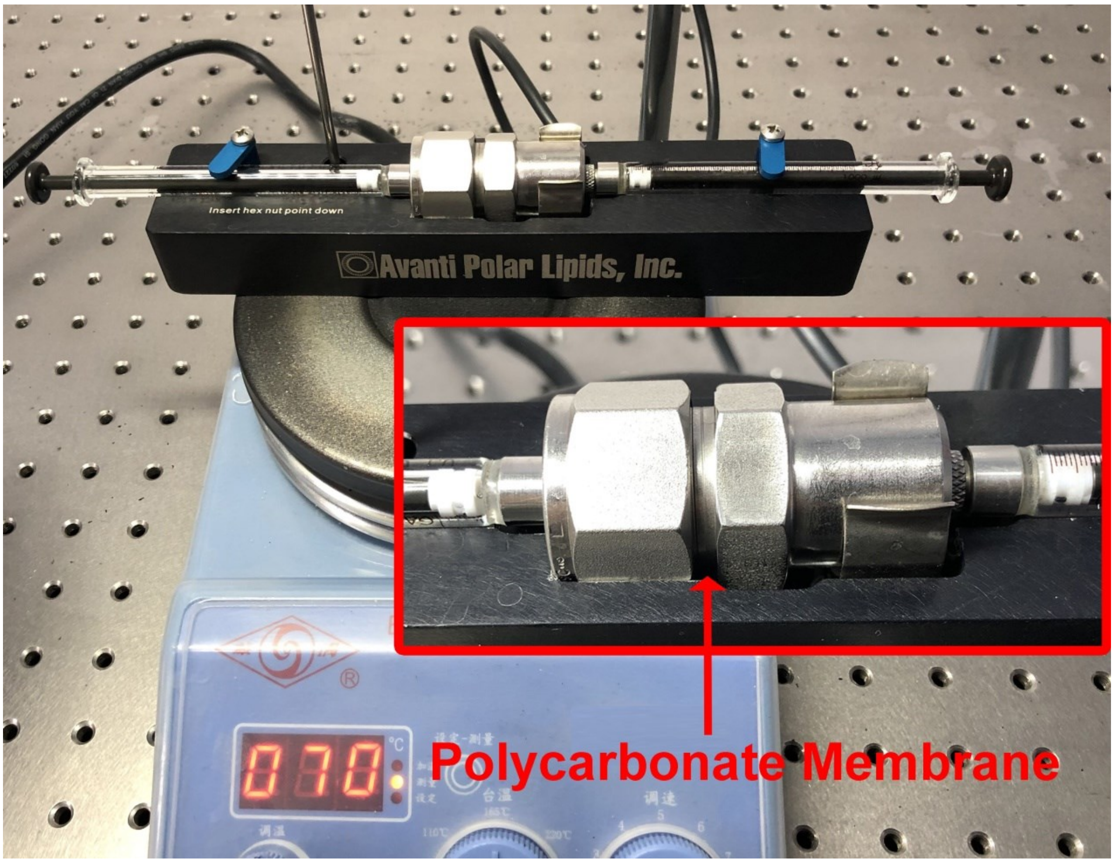

Fig. S1 Small-volume extrusion apparatus for preparing liposomes with a fixed uniform size. The red arrow points to the location of the polycarbonate membrane.

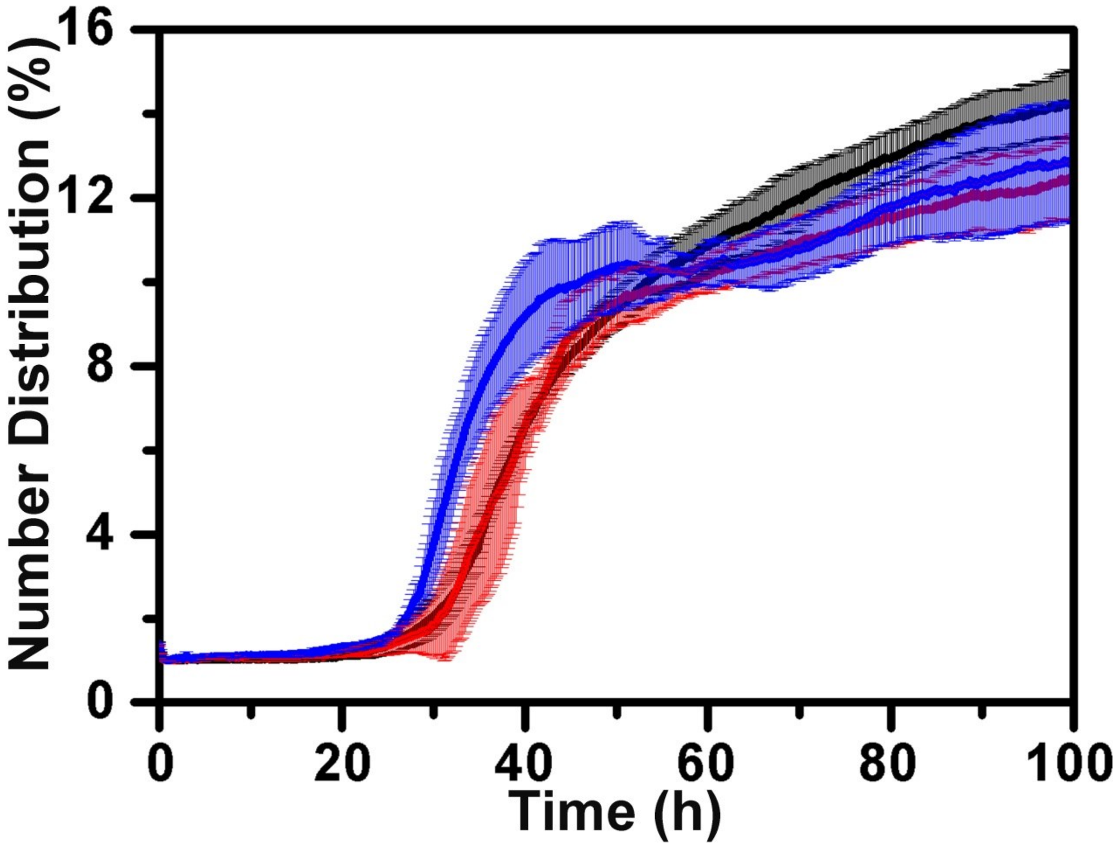

Fig. S2 ThT monitored kinetic curves for fibril formation of Aβ(1−40) monomers at 37 °C: Aβ(1−40) alone (black); Aβ(1−40) with L-Asp (red) or D-Asp (blue) added. The fibrillation of Aβ(1−40) was not affected obviously when L- or D-Asp was added individually. In all panels, error bars show the standard deviations of the averaged data sets. Experiments were repeated three times.

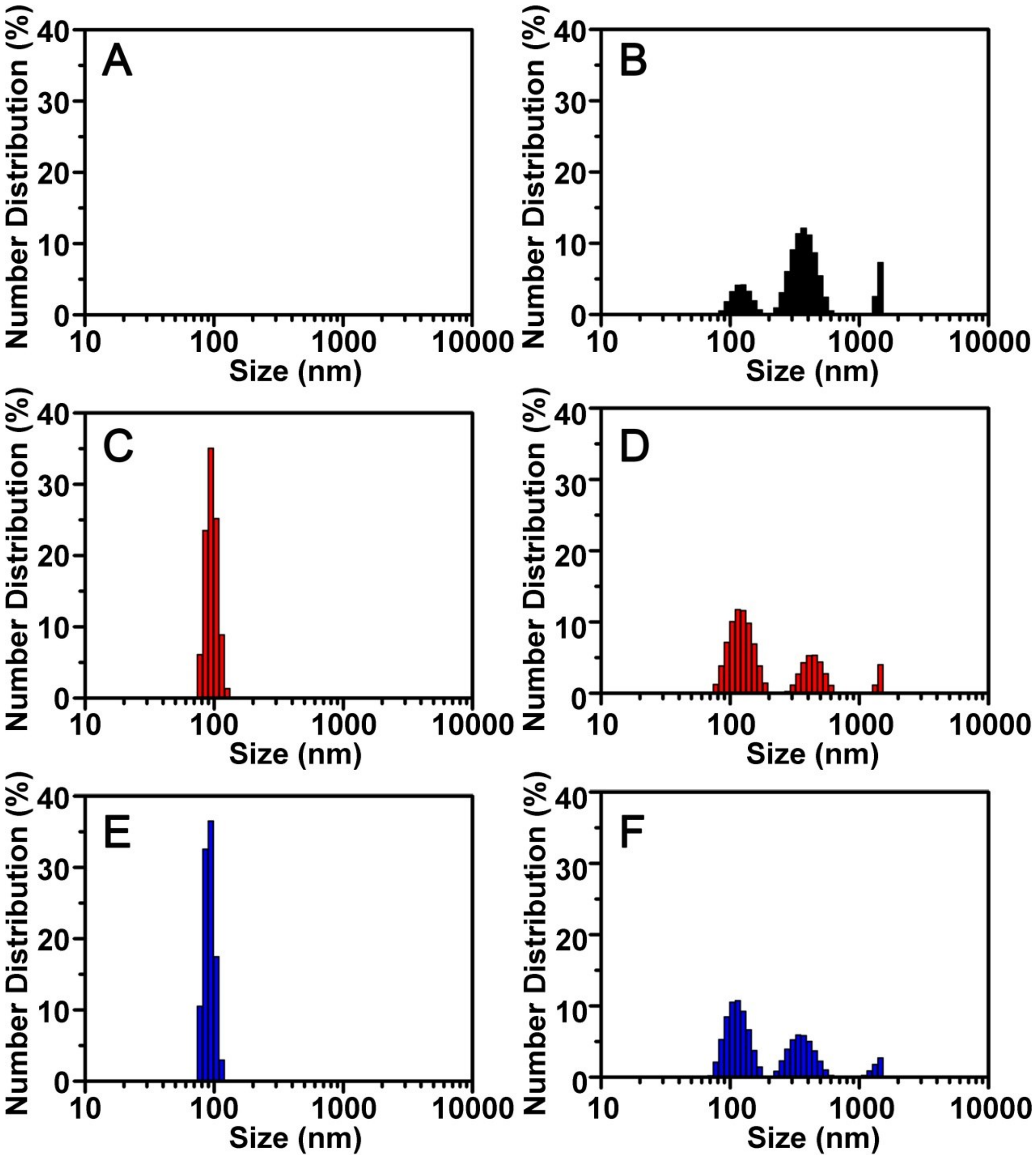

Fig. S3 Hydrodynamic radius distribution: (A) Aβ(1−40) monomers alone. (C, E) newly extruded L- (C) or D-Asp-DPPE liposomes (E). (B, D, F) Aβ(1−40) monomers alone (B), Aβ(1−40) with L- (D) or D-Asp-DPPE liposomes (F) added after incubation at 37 °C for 80 h. The size of the chiral liposomes was slightly increased. Compared to Aβ(1−40) added chiral liposomes, pure Aβ(1−40) obtained more long fibers after incubation.

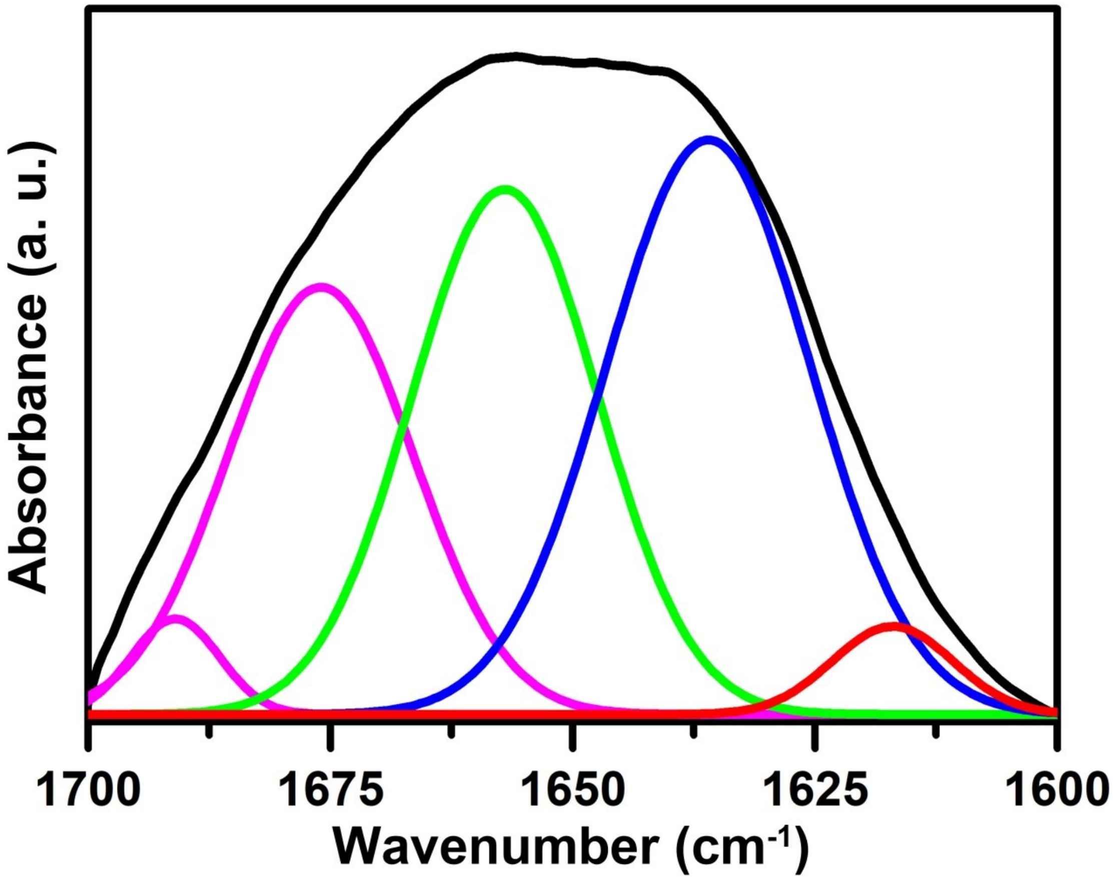

Fig. S4 Original and fitting amide I FT-IR spectra of Aβ(1−40) (25 μM). The red, blue, green and pink curves in the figure represent β-sheet, random coil, α-helix and β-turn structure, respectively.

Table S1 Quantitative estimation of the secondary structures of Aβ(1−40). Data are shown as the mean ± SD (n = 3).

**Secondary structure**	α-helix	β-sheet	β-turn	Random coil
**Proportion (%)**	29 ± 2	6 ± 2	27 ± 1	38 ± 1

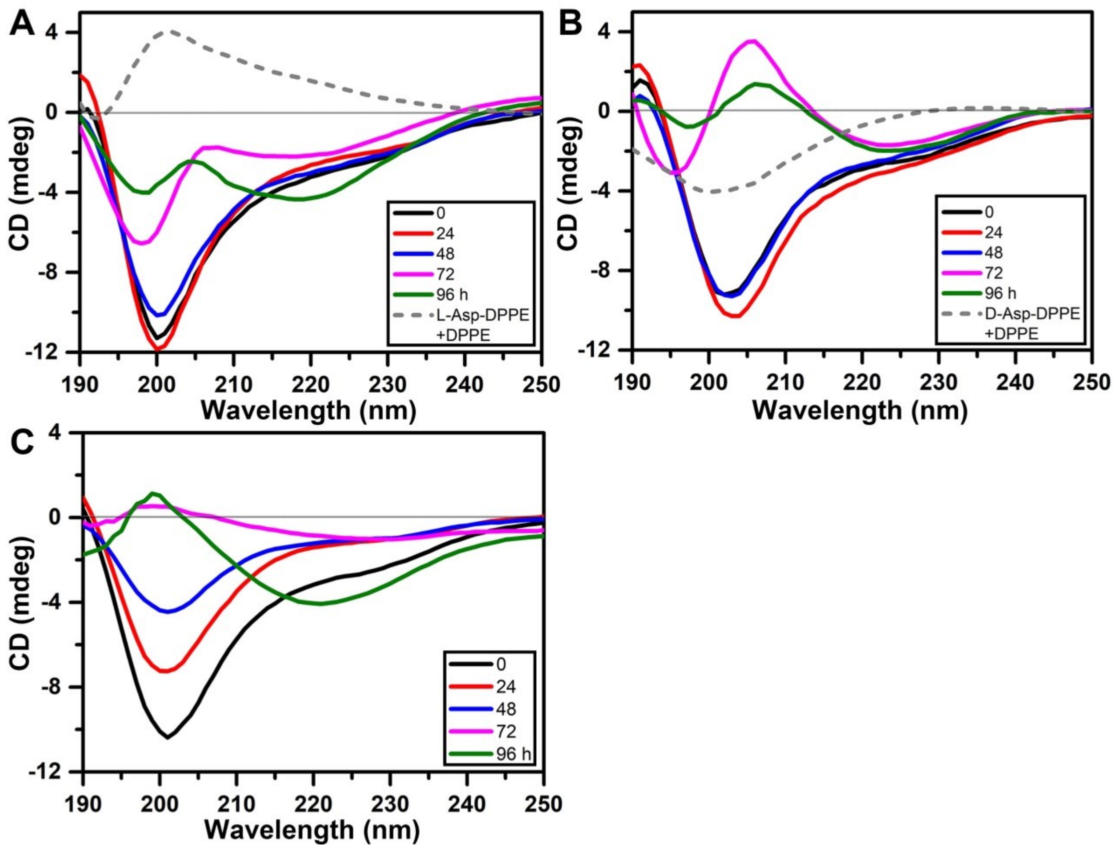

Fig. S5 Time dependence of CD spectra changes of Aβ(1−40) with the addition of L- (A) or D-Asp-DPPE (B) liposomes, or Aβ(1−40) alone (C) during fibrillogenesis at 37 °C. The final concentrations of Aβ(1−40) and chiral liposomes are 25 μM and 0.5 mg·mL^{-1}, respectively.

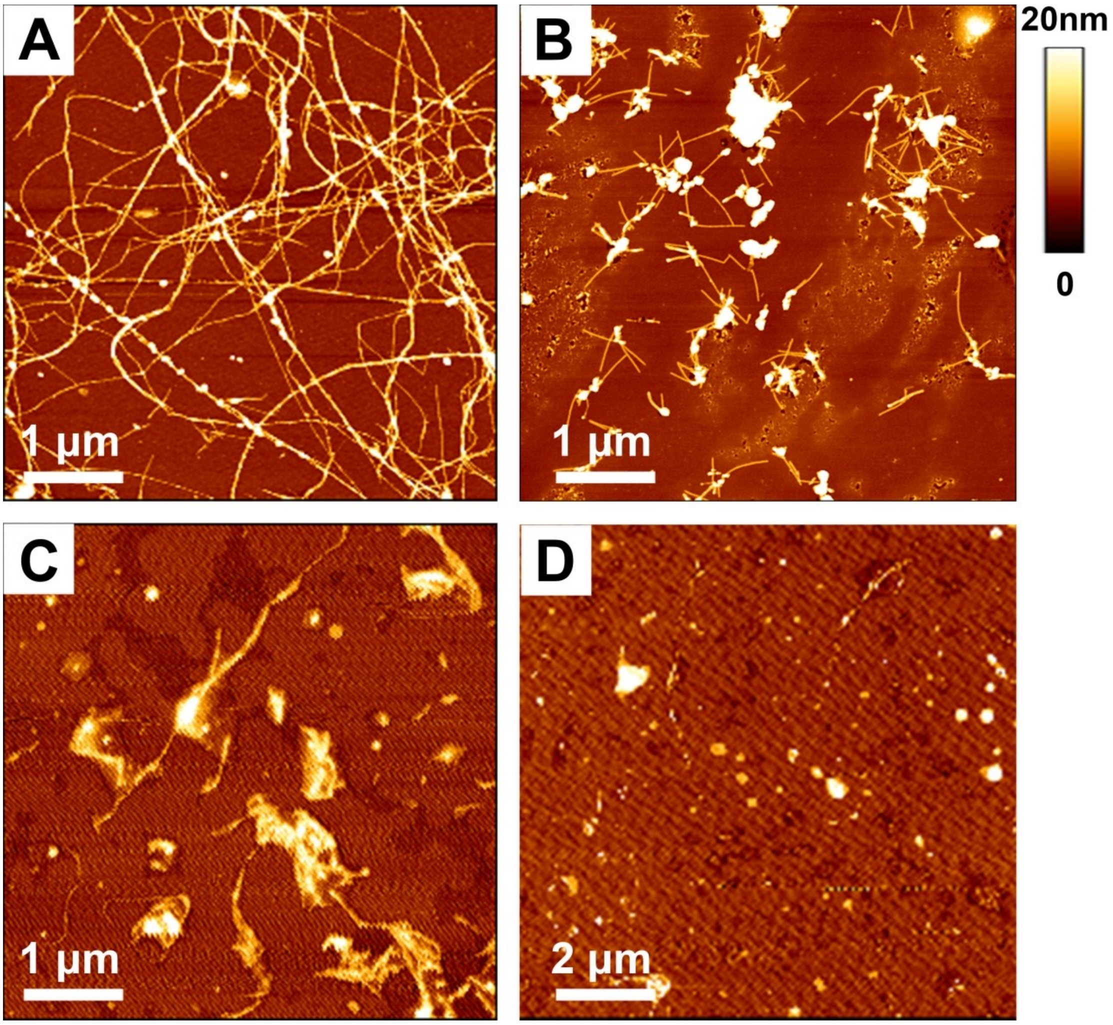

Fig. S6 AFM images obtained after incubation of Aβ(1−40) with liposomes at 37 °C for 80 h. (A) Aβ(1−40) alone. (B−D) Aβ(1−40) with DPPE (B), L-Asp-DPPE (C), or D-Asp-DPPE (D) liposomes added.

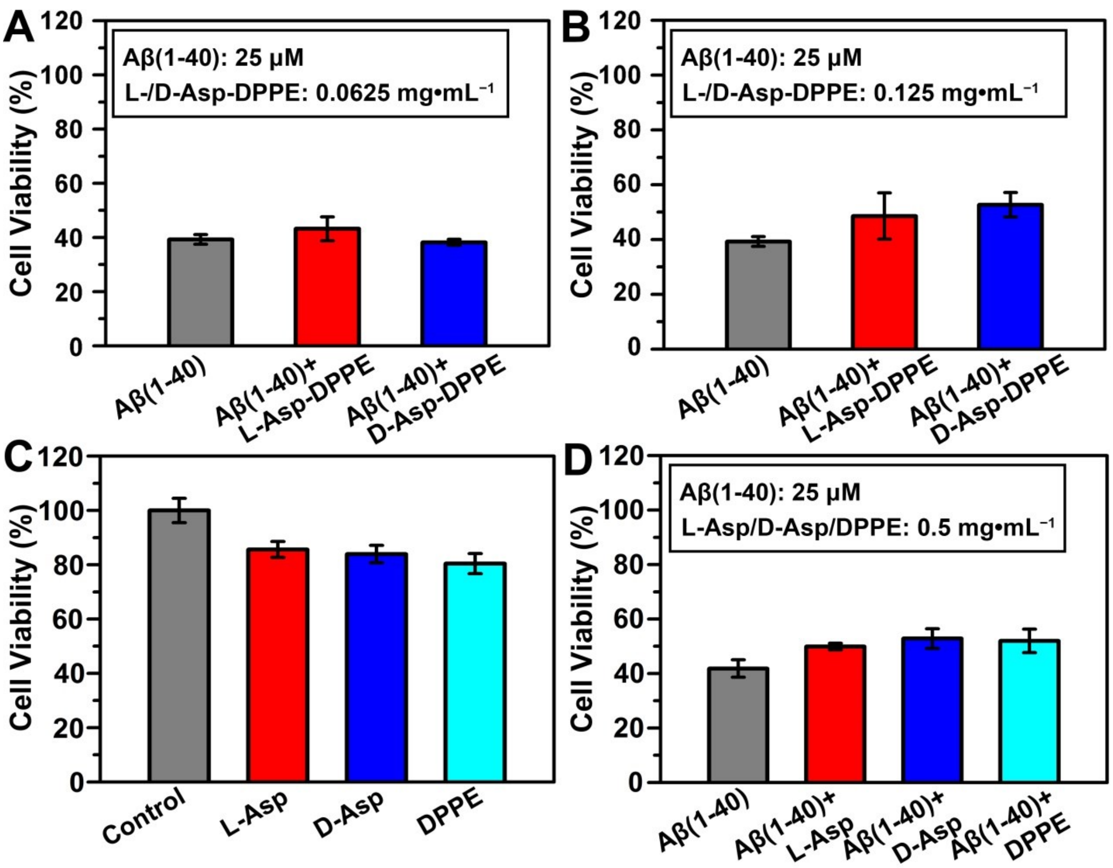

Fig. S7 Cell viability test. (A, B) Effects of L- or D-Asp-DPPE liposomes on Aβ(1−40)–aggregation induced cytotoxicity. (C) Effects of L-Asp, D-Asp, or DPPE liposomes on cell viability. (D) Effects of L-Asp, D-Asp, or DPPE liposomes on Aβ(1−40)–aggregation induced cytotoxicity. The concentration of Aβ(1−40) is 25 μM. The concentrations of L- and D-Asp-DPPE liposomes are 0.0625 mg·mL^{-1} (A) and 0.125 mg·mL^{-1} (B), respectively. The concentration of L-Asp, D-Asp, and DPPE liposomes are 0.5 mg·mL^{-1}. Each experiment had been repeated three times. Error bars indicate ±s.d.

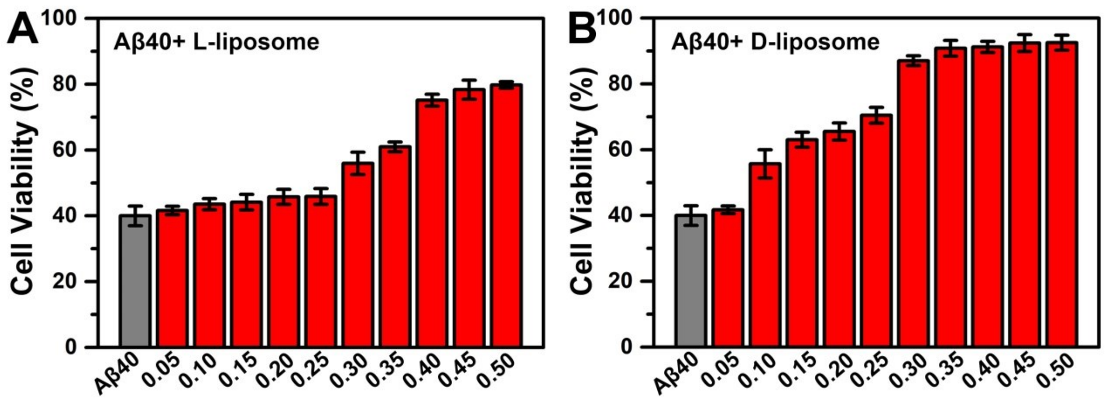

Fig. S8 Cytotoxicity of 25 μM Aβ(1−40) applied to N2a cells in the absence or presence of L-

(A) or D-Asp-DPPE liposomes (B) at the indicated concentrations (from 0.05 mg·mL^{-1} to 0.50 mg·mL^{-1}). Each experiment had been repeated three times. Error bars indicate ±s.d.

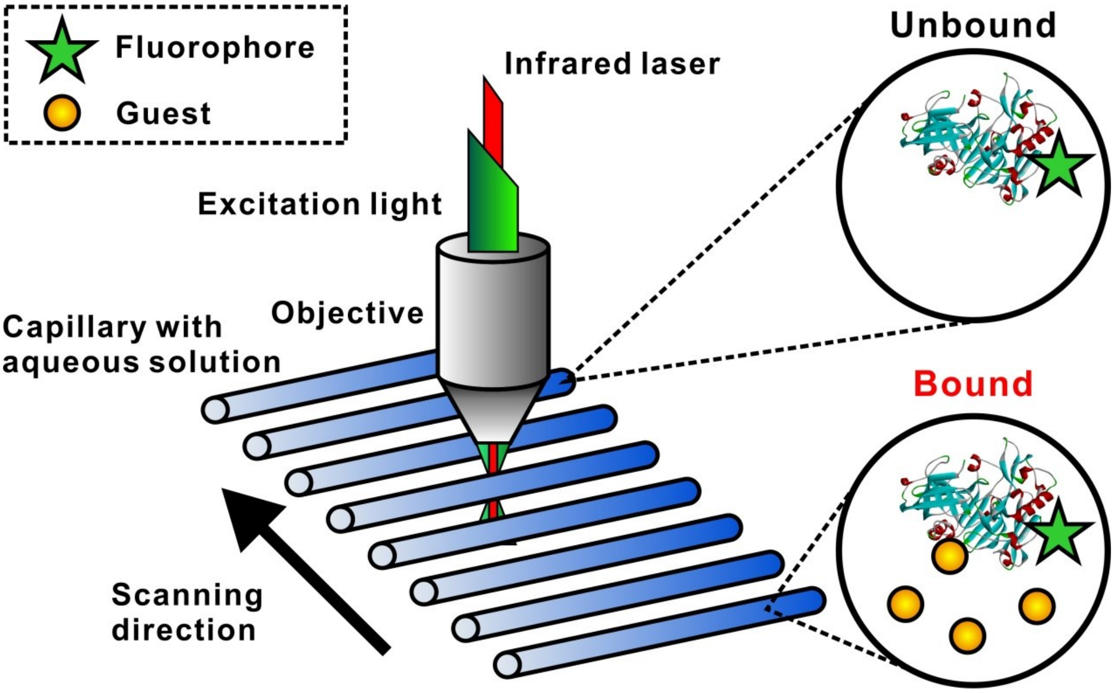

Fig. S9 Technical setup of the MST. Optics, focused in the center of the glass capillary, record the thermophoretic movement of the optical visible molecule through temperature gradients that are established by an IR laser.

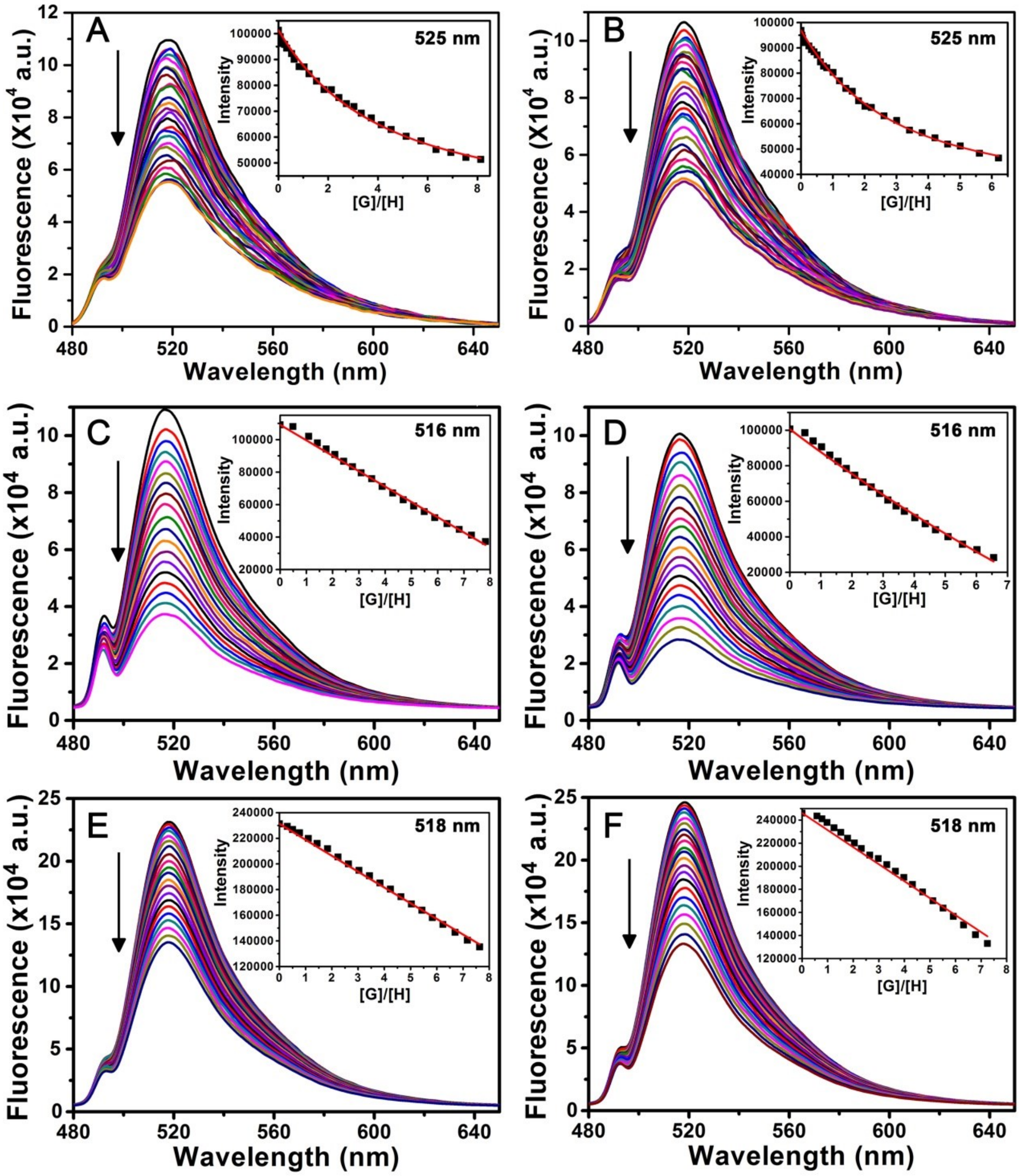

Fig. S10 Fluorescence spectra of fluorescein–labelled oligopeptides (i.e., VHHQKLVFF (A, B), LVFF (C, D), VHHQ (E, F)) (5.0×10^{-6} mol·L^{-1}) upon addition of different equivalents of PEA L- or D-Asp in H_2O containing 0.2% DMSO at 25 °C. The insets show the fluorescent intensity changes of host oligopeptides upon the additions of various guests, [G]/[H] is an abbreviation of the molar ratio of guest to host. The red lines are nonlinear fitting curves.

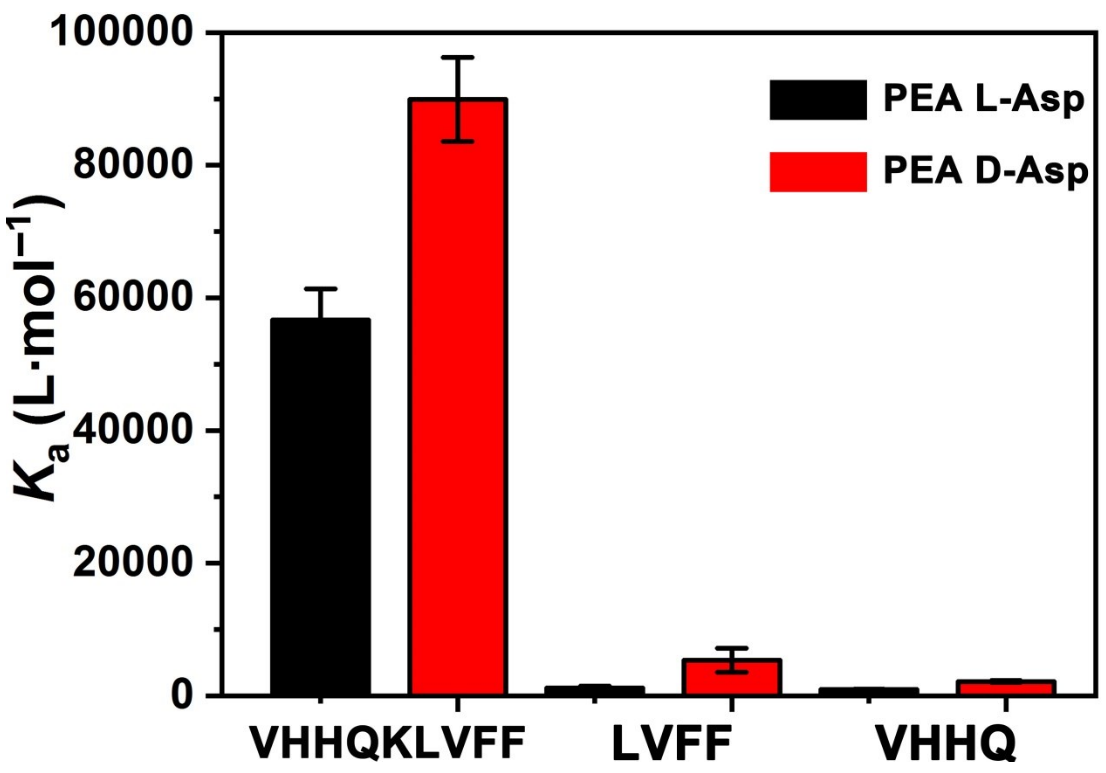

Fig. S11 The association constant (K_a) of fluorescein–labelled oligopeptides (i.e., VHHQKLVFF, LVFF, and VHHQ) with PEA L- or D-Asp in the fluorescence titration experiment. In H_2O solution containing 0.2% DMSO, the K_a of VHHQKLVFF with PEA L-Asp, PEA D-Asp was 56684 and 89920 L·mol^{-1}, respectively. The K_a of LVFF with PEA L-Asp, PEA D-Asp was 1158 and 5344 L·mol^{-1}, respectively. The K_a of VHHQ with PEA L-Asp, PEA D-Asp was 968 and 2142 L·mol^{-1}, respectively.

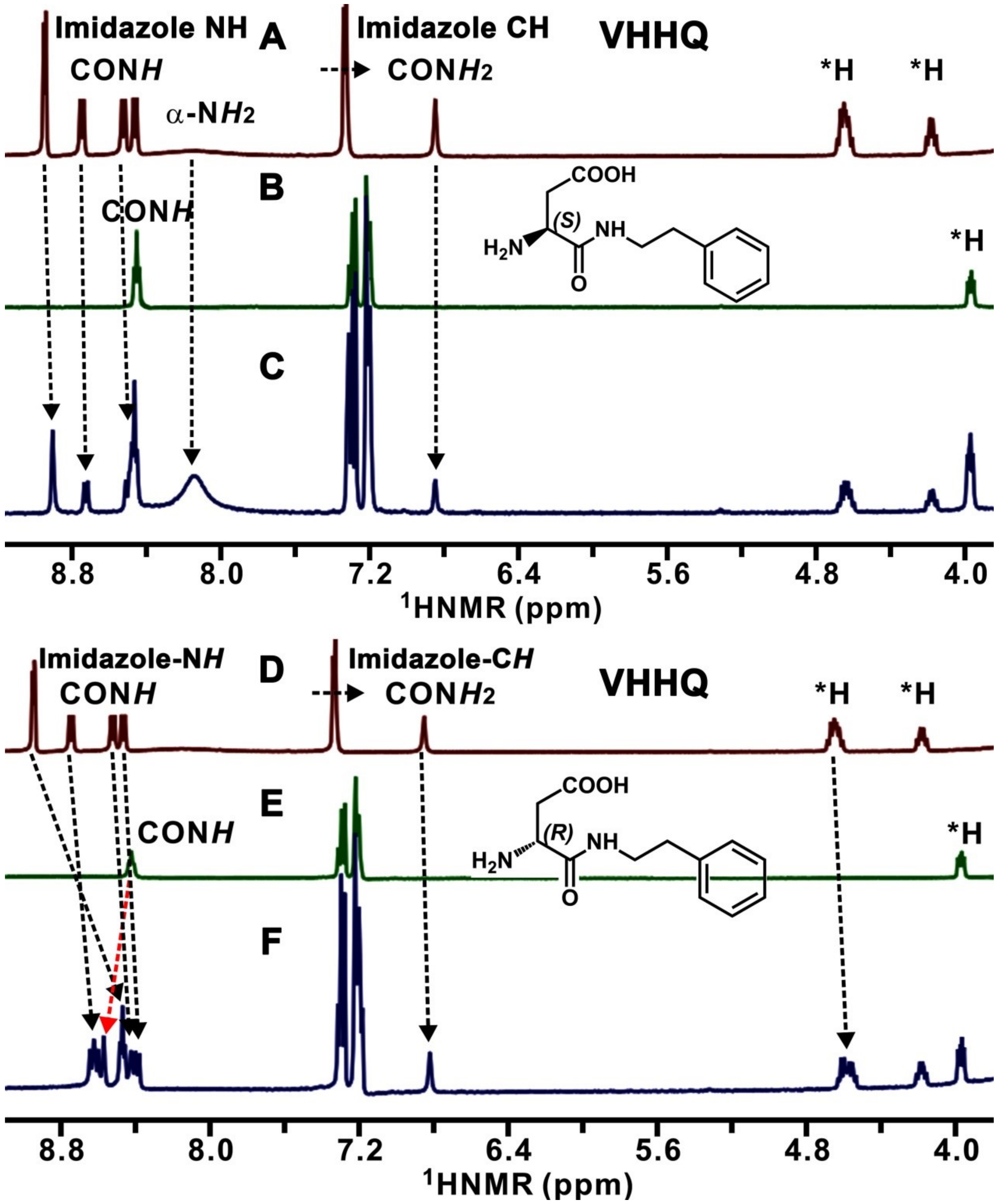

Fig. S12 Parts of the ^{1}H NMR spectra: (A−C) VHHQ (A), PEA L-Asp (B), and mixtures of VHHQ with equimolar amount of PEA L-Asp (C) in d_6-DMSO at 20 °C. (D−F) VHHQ (D), PEA D-Asp (E), and mixtures of VHHQ with equimolar amount of PEA D-Asp (F) in d_6-DMSO at 20 °C. Chemical shift changes of active H–protons are indicated by black arrows.

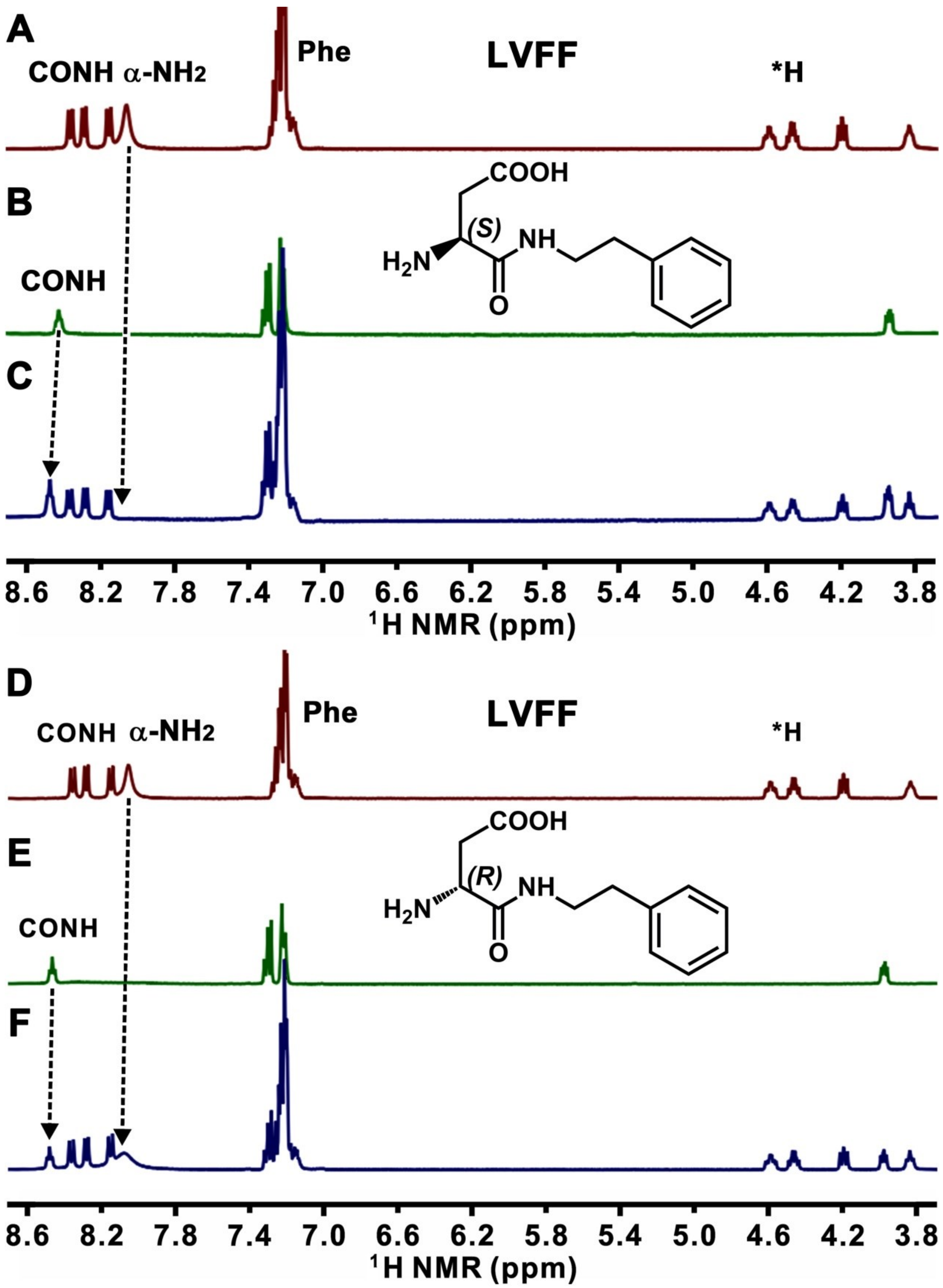

Fig. S13 Parts of the ^{1}H NMR spectra: (A−C) LVFF (A), PEA L-Asp (B), and mixtures of LVFF with equimolar amount of PEA L-Asp (C) in d_6-DMSO at 20 °C. (D−F) LVFF (D), PEA D-Asp (E), and mixtures of LVFF with equimolar amount of PEA D-Asp (F) in d_6-DMSO at 20 °C. Chemical shift changes of active H–protons are indicated by black arrows.

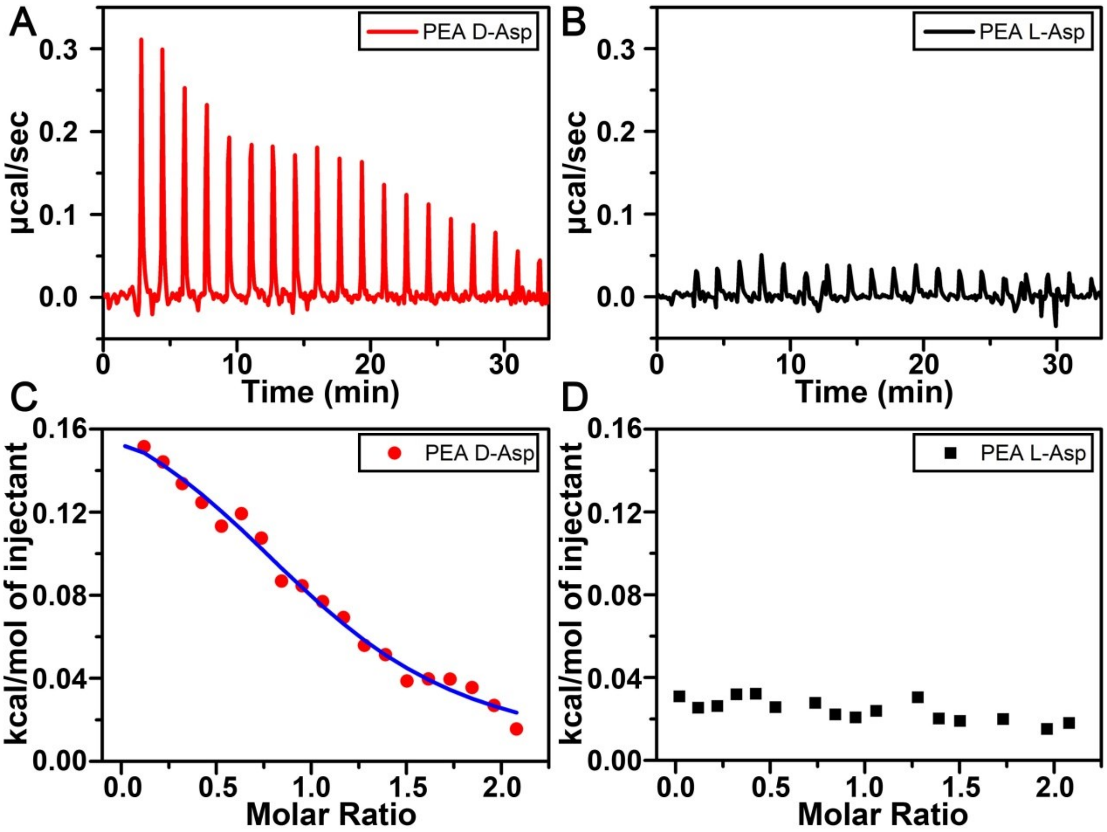

Fig. S14 Isothermal titration calorimetry data and fitting curve of PEA D-Asp (A, C) and PEA L-Asp (B, D) (10 mM) titrated into VHHQKLVFF (1 mM) in H_2O containing 5% DMSO at 25 °C. The K_a of VHHQKLVFF with PEA D-Asp was 3590 L·mol^{-1}. The influence of DMSO had been subtracted through background correction.

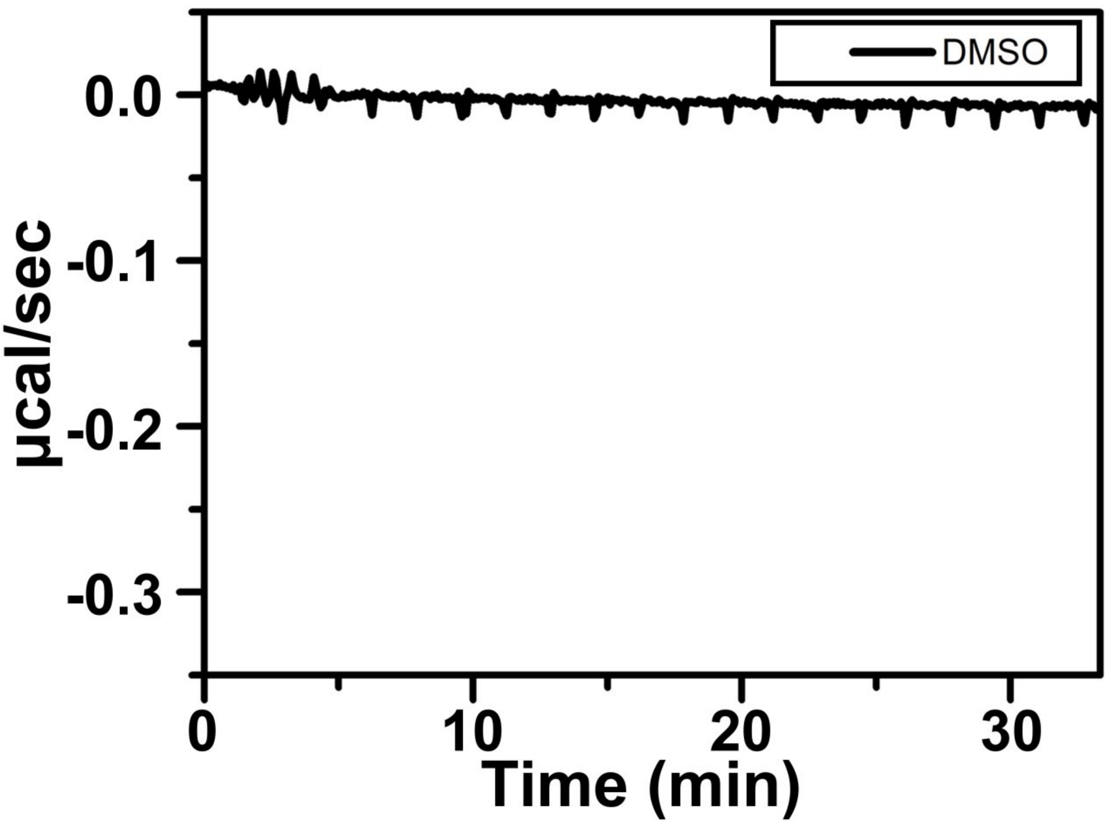

Fig. S15 Isothermal titration calorimetry data using H_2O containing 5% DMSO as titrant and the titrand at 25 °C, which was used for background correction of Fig. S14.

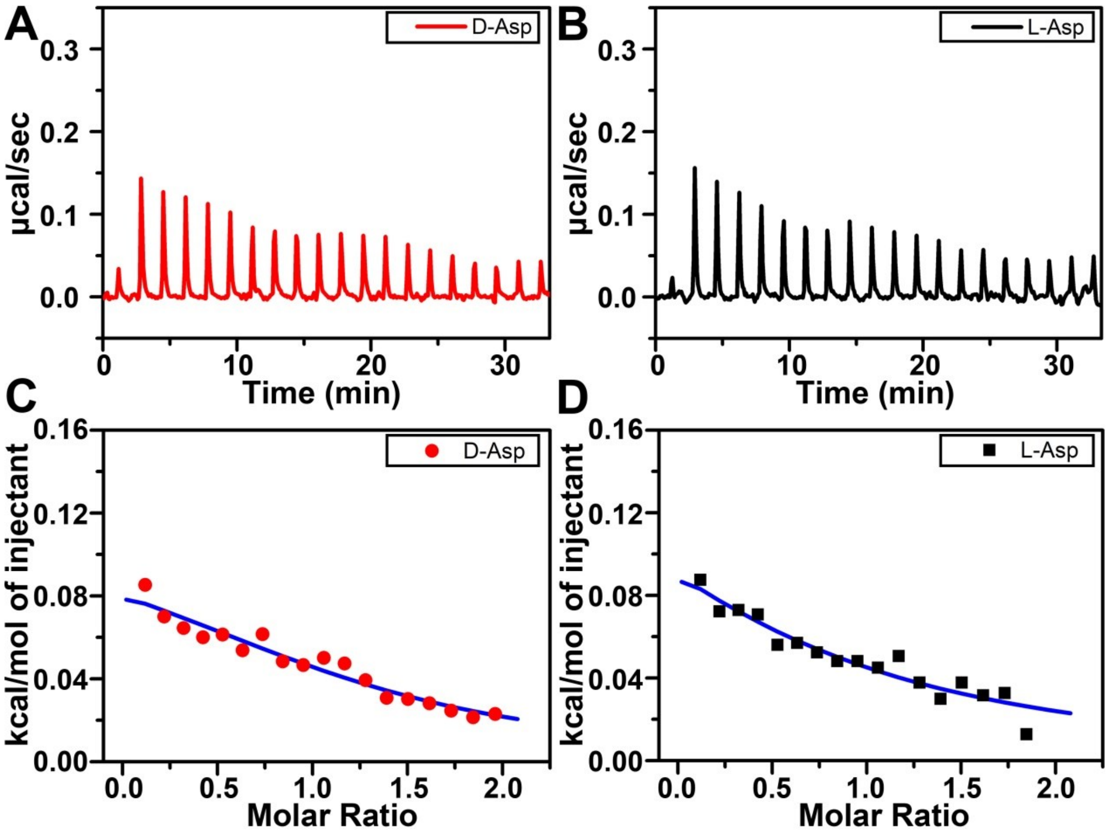

Fig. S16 Isothermal titration calorimetry data and fitting curve of D-Asp (A, C) and L-Asp (B, D) (10 mM) titrated into VHHQKLVFF (1 mM) in H_2O at 25 °C. The K_a of VHHQKLVFF with D-Asp, L-Asp was 1570 L·mol^{-1} and 691 L·mol^{-1}, respectively.

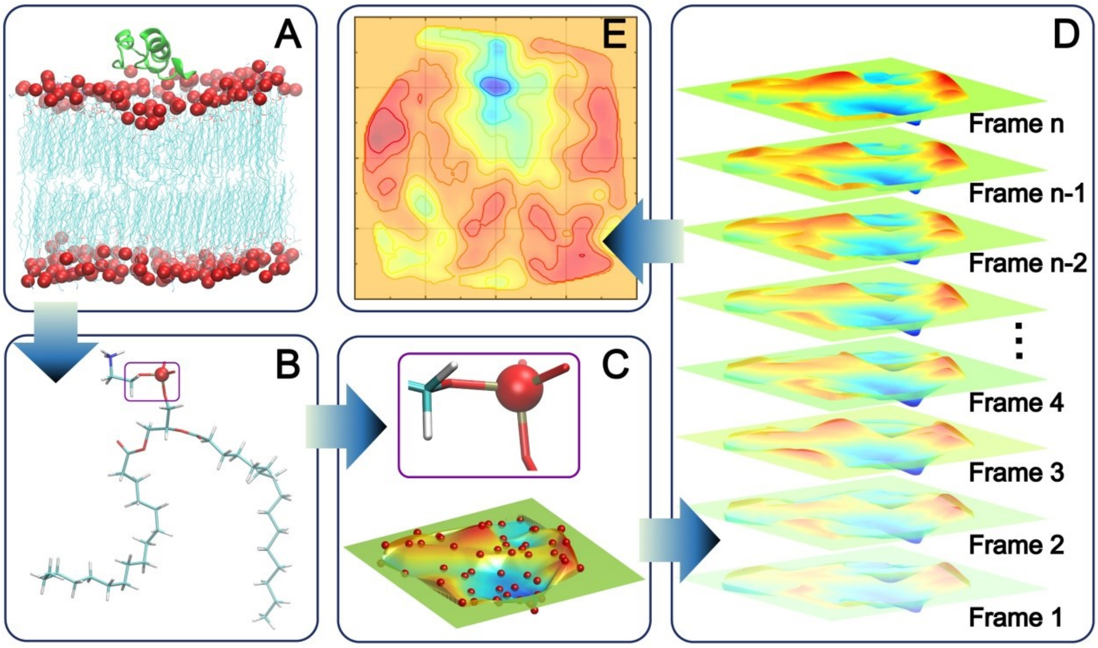

Fig. S17 The illustration of fitting the P_z surface of the equilibrium trajectory. (A) A model of

D-Asp-DPPE bilayer with Aβ(1−40). (B) One of the D-Asp-DPPE phospholipids is shown as sticks model. (C) The PO_4 atoms and a smooth version of P_z surface of the first frame. (D) The P_z surface in each frame of MD trajectory. (E) The mean smooth P_z surface of trajectory in equilibrium state.

Supplementary References

(1) G. Y. Qing, S. L. Zhao, Y. T. Xiong, Z. Y. Lv, F. L. Jiang, Y. Liu, H. Chen, M. X. Zhang, T. L. Sun, *J. Am. Chem. Soc.* 2014, **136**, 10736–10742.

(2) J. Yang, X. L. Zhang, Y. Y. Zhu, E. Lenczowski, Y. L. Tian, J. Yang, C. Zhang, M. Hardt, C. H. Qiao, R. E. Tanzi, A. Moore, H. Ye, C. Z. Ran, *Chem. Sci.* 2017, **8**, 6155−6164.

(3) G. B. Gao, M. X. Zhang, D. J. Gong, R. Chen, X. J. Hu, T. L. Sun, *Nanoscale* 2017, **9**, 4107−4113.

(4) S. Kumar, A. Henning-Knechtel, M. Magzoub, A. D. Hamilton, *J. Am. Chem. Soc.* 2018, **140**, 6562−6574.

(5) R. C. MacDonald, R. I. MacDonald, B. P. M. Menco, K. Takeshita, N. K. Subbarao, L. R. Hu, *Biochim. Biophys. Acta, Biomembr.* 1991, **1061**, 297–303.

(6) B. Mui, L. Chow, M. J. Hope, *Meth. Enzymol.* 2003, **367**, 3–14.

(7) J. T. Pelton, L. R. McLean, *Anal. Biochem.* 2000, **277**, 167–176.

(8) Y. P. Dai, M. X. Zhang, X. L. Shi, K. Wang, G. B. Gao, L. Shen, T. L. Sun, *Colloids Surf., B* 2020, **185**, 110615.

(9) M. Richman, S. Wilk, M. Chemerovski, S. K. T. S. Wärmländer, A. Wahlström, A. Gräslund, S. Rahimipour, *J. Am. Chem. Soc.* 2013, **135**, 3474–3484.

(10) A. Wahlström, L. Hugonin, A. Perálvarez-Marín, J. Jarvet, A. Gräslund, *FEBS J.* 2008, **275**, 5117–5128.

(11) S. Vivekanandan, J. R. Brender, S. Y. Lee, A. Ramamoorthy, *Biochem. Biophys. Res. Commun.* 2011, **411**, 312–316.

(12) M. J. Frisch, G. W. Trucks, H. B. Schlegel, G. E. Scuseria, M. A. Robb, J. R. Cheeseman, G. Scalmani, V. Barone, B. Mennucci, G. A. Petersson, H. Nakatsuji, M. Caricato, X. Li, H. P. Hratchian, A. F. Izmaylov, J. Bloino, G. Zheng, J. L. Sonnenberg, M. Hada, M. Ehara, K. Toyota,

R. Fukuda, J. Hasegawa, M. Ishida, T. Nakajima, Y. Honda, O. Kitao, H. Nakai, T. Vreven, J. A. Montgomery Jr, J. E. Peralta, F. Ogliaro, M. Bearpark, J. J. Heyd, E. Brothers, K. N. Kudin, V. N. Staroverov, T. Keith, R. Kobayashi, J. Normand, K. Raghavachari, A. Rendell, J. C. Burant, S. S. Iyengar, J. Tomasi, M. Cossi, N. Rega, J. M. Millam, M. Klene, J. E. Knox, J. B. Cross, V. Bakken, C. Adamo, J. Jaramillo, R. Gomperts, R. E. Stratmann, O. Yazyev, A. J. Austin, R. Cammi, C. Pomelli, J. W. Ochterski, R. L. Martin, K. Morokuma, V. G. Zakrzewski, G. A. Voth, P. Salvador, J. J. Dannenberg, S. Dapprich, A. D. Daniels, Ö. Farkas,J. B. Foresman, J. V. Ortiz, J. Cioslowski, D. J. Fox, 2009.

(13) D. Case, R. Betz, D. S. Cerutti, T. Cheatham, T. Darden, R. Duke, T. J. Giese, H. Gohlke, A. Götz, N. Homeyer, S. Izadi, P. Janowski, J. Kaus, A. Kovalenko, T. S. Lee, S. LeGrand, P. Li, C. Lin, T. Luchko, P. A. Kollman, *Amber 2016, University of California, San Francisco*, 2016.

(14) R. W. Hockney, S. P. Goel, J. W. Eastwood, *J. Comput. Phys.* 1974, **14**, 148–158.

(15) T. Darden, D. York, L. Pedersen, *J. Chem. Phys.* 1993, **98**, 10089–10092

(16) P. J. Rossky, J. D. Doll, H. L. Friedman, *J. Chem. Phys.* 1978, **69**, 4628–4633.

(17) M. J. Janiak, D. M. Small, G. G. Shipley, *Biochemistry* 1976, **15**, 4575–4580.

(18) J. A. Izaguirre, D. P. Catarello, J. M. Wozniak, R. D. Skeel, *J. Chem. Phys.* 2001, **114**, 2090–2098.

(19) S. Leekumjorn, A. K. Sum, *Biochim. Biophys. Acta, Biomembr.* 2007, **1768**, 354–365.

(20) D. R. Roe, T. E. Cheatham, *J. Chem. Theory Comput.* 2013, **9**, 3084–3095.

(21) R. C. Gonzalez, R. E. Woods, *Digital Image Processing (3rd Edition)*; Prentice-Hall, Inc., 2006.

(22) R. J. Falconer, *J. Mol. Recognit.* 2016, **29**, 504–515.
